# Biosensor Approach to Psychopathology Classification

**DOI:** 10.1371/journal.pcbi.1000966

**Published:** 2010-10-21

**Authors:** Misha Koshelev, Terry Lohrenz, Marina Vannucci, P. Read Montague

**Affiliations:** 1 Program in Cell and Molecular Biology, Baylor College of Medicine, Houston, Texas, United States of America; 2 W. M. Keck Center for Interdisciplinary Bioscience Training, Houston, Texas, United States of America; 3 Department of Neuroscience, Computational Psychiatry Unit, Baylor College of Medicine, Houston, Texas, United States of America; 4 Department of Statistics, Rice University, Houston, Texas, United States of America; John Radcliffe Hospital, United Kingdom

## Abstract

We used a multi-round, two-party exchange game in which a healthy subject played a subject diagnosed with a DSM-IV (Diagnostic and Statistics Manual-IV) disorder, and applied a Bayesian clustering approach to the behavior exhibited by the healthy subject. The goal was to characterize quantitatively the style of play elicited in the healthy subject (the proposer) by their DSM-diagnosed partner (the responder). The approach exploits the dynamics of the behavior elicited in the healthy proposer as a biosensor for cognitive features that characterize the psychopathology group at the other side of the interaction. Using a large cohort of subjects (*n* = 574), we found statistically significant clustering of proposers' behavior overlapping with a range of DSM-IV disorders including autism spectrum disorder, borderline personality disorder, attention deficit hyperactivity disorder, and major depressive disorder. To further validate these results, we developed a computer agent to replace the human subject in the proposer role (the biosensor) and show that it can also detect these same four DSM-defined disorders. These results suggest that the highly developed social sensitivities that humans bring to a two-party social exchange can be exploited and automated to detect important psychopathologies, using an interpersonal behavioral probe not directly related to the defining diagnostic criteria.

## Author Summary

Human social interaction is exquisitely complex, and perturbed social interaction is a hallmark of psychological pathogy. When someone has a psychological disorder the focus is generally on their behavior, but this behavior is rarely something displayed in isolation and typically induces profound changes in the people interacting with the disturbed individual. In this work we asked if the behavior of one person in a simple two-person economic exchange game is sensitive to features that could classify the pathology of their partner. We analyzed a large group of previously recorded interactions involving healthy persons and people diagnosed with a variety of psychological disorders, and found that a healthy person's behavior is indeed quantitatively and systematically influenced by their partner's pathology. These results could ultimately lead to a different way of understanding and diagnosing psychological disease.

## Introduction

### Fairness games as probes for social exchange

Social interactions among humans reflect the execution of some of the most important and complex behavioral software with which humans are endowed. Consequently, we should expect the computations involved in human social exchange to be subtle and perhaps even difficult to expose and study in controlled settings. However, exposing these computations is crucial if we are to improve our characterization and understanding of normal human cognitive function and dysfunction.

In recent years, the components of social exchange in healthy subjects have been probed using interactive economic exchange games [1]–[8]. These games typically involve two subjects interacting for one or multiple rounds through the exchange of monetary gestures to one another. For our purposes here, these games require three classes of computation be intact and functioning in the minds of the interacting subjects. They require that each subject can (1) compute norms for what is fair in each exchange, (2) detect deviations in monetary gestures that deviate from these norms, and (3) choose actions predicated on such deviations [9]–[15]. These experimental probes have been used previously in the area of behavioral economics and neuroeconomics, but here we show that the behavioral gestures elicited in the context of economic exchange games can be used to classify certain psychopathologies. The twist in our effort here is that we use a data-driven approach examining the reactions of the healthy partner as a kind of biosensor while playing an exchange game with a subject possessing a psychopathology.

### Multi-round trust game

**Figure 1:**
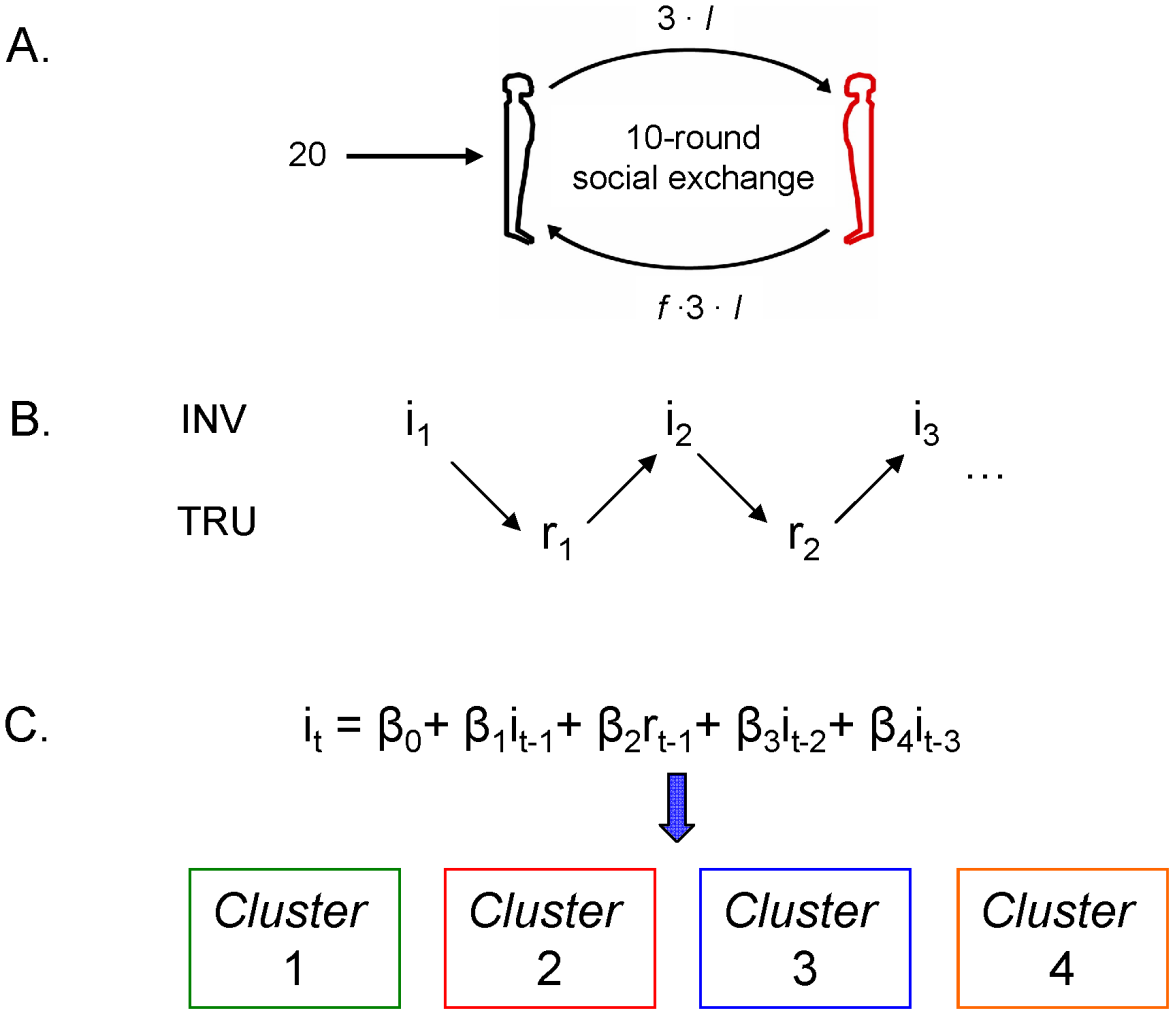
Model-free clustering of an objective multi-round economic exchange game. A) Depiction of Multi-Round Trust Task. A ten round task in which two players, an investor and a trustee, undergo repeated interactions. Adapted from previous publications [6], [7], [16], [20]. B&C) Our Approach. Following [23], we cluster investor-trustee dyads based on a regression of previous choices in the trust game. Specifically, we predict ratios of investment *i_t_* in round *t* as a polynomial of past rounds of investment and return. The number of clusters, order of polynomial, and number of rounds back on which to base this dependence are all taken as free parameters in the model.

In this paper, we used a multi-round fairness game played by pairs (“dyads”) of interacting humans to extract behavioral phenotypes defined by the dynamics of play exhibited over the 10 rounds of a complete game [6], [7], [16]. The game we employ is called a *trust game* [17]–[19]; see Figure 1A. In the 10-round trust game, one player (called the *investor* or the *proposer*) is endowed with 20 monetary units and chooses to send some fraction 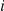 to their partner (called the *trustee* or the *responder*). The amount sent is tripled to 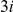 on the way to the trustee. The trustee decides which fraction 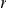 to return in response to the investor, thus each round is represented by two numbers: the investment fraction 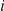 and the repayment fraction 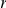. All the rules are transparent to both players. The game is played for 10 rounds and the repeated exchanges allow the players to build models of what to expect from their partner providing that their capacity to sense, model, and respond to their partner's decisions is intact.

In most of the dyads, the subjects were given no information about their partner and did not meet or speak to the partner before, during, or after the task. Following [16], we also included “personal” dyads, in which the partners met before the task, were instructed together, and saw a picture of their partner during each round.

### Biosensor hypothesis

The basic approach of this paper derives from our prior work showing that this same game elicits unique behavioral phenotypes when a game is played between a healthy investor and a trustee diagnosed with a range of DSM-defined disorders – Autism Spectrum Disorder (ASD) [20], Borderline Personality Disorder (BPD) [6], Major Depressive Disorder (MDD), and Attention Deficit Hyperactivity Disorder (ADHD) [20]. In all these studies, we noticed that the behavioral differences affect not only the trustee, but also a healthy investor who plays with this trustee.

A similar conclusion that a healthy subject is sensing the psychological nature of the opponent during play was obtained in a recent paper [21], where it was shown that a subject can gauge the strategic sophistication of the opponent in repeated play of a complex stag hunt game.

These observations suggested the hypothesis that the healthy (or typical) investor's behavior might be used to ‘read out’ features that could characterize the psychopathology group playing in the trustee role. This possibility was also suggested by the nature of the interpersonal interaction enforced by the game. In any multi-round interaction with another human, a player's choices are rather dramatically entangled with those of her partner. In addition, although the game is characterized by two numbers per exchange (investment and repayment ratio), it does require players to have several cognitive capacities intact to accomplish a ‘normal’ exchange. These include short-term and working memory, sufficiently accurate models of what to expect from another human in this exchange, appropriate sensitivity to positive and negative social signals, and intact capacity to respond to such signals. Collectively, these observations support the basic hypothesis that humans bring highly developed social sensitivities to two-party interactions that might be profitably exploited as a biological “sensor” (*biosensor*) – first using a human proposer (investor) and later capturing this behavior in a computer agent.

## Results

### Available data

We analyze the results of 287 dyads, in which healthy participants play against healthy trustees, as well as against the trustees that have four different disorders: ASD, BPD, MDD, and ADHD. Each subject played only one game. With the exception of some patients with BPD, participants with disorders were not medicated. A detailed description of the data is given in Table S1.

### Bayesian classification of multi-round social exchange

We sought to classify the dynamics using only the numbers exchanged in the game between players (investment and repayment ratios), the number of “types” or styles of play (number of clusters), and the functional dependence of the next investment on preceding investment and repayment ratios. In short, we sought a heavily data-driven approach.

We extended a previously published method [22], [23] to cluster available trust game data. This method uses a regression approach to the functional dependence that clusters individuals based on coefficients of the regression. This method has advantages over traditional clustering approaches: (i) the number of types in our population is estimated directly from the data, and (ii) classification uncertainty is captured by probabilities rather than categorical cluster assignments. An investor is not classified as either within or not within a cluster, but instead a probability of being in a cluster is computed. This allows us to identify clusters where a style of behavior (a type) is over-represented (in comparison with what is expected by chance), under-represented, or neither (see below for details of this calculation).

### Data-driven modeling of two-party social exchange in the trust game

The basic model is determined directly from the numbers exchanged by the two players during the game. We model the healthy proposer's investment at time 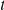 as a function of preceding investment and repayment ratios. In this “black-box”, regression approach [22]–[23], we assume that we can capture meaningful variations in types of investor play by using a regression model based only on previous investment and return ratios, in contrast to other approaches [21], [24] which commit to more explicit models of how these values are used in mental processes to generate behavior.

It is known that an arbitrary continuous function can be approximated, with any given accuracy, by a polynomial of an appropriate order. As a result, a widely used approach to describe such functions is to try polynomial dependence of increasing order. For a first order dependence of the current investment 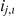 on previous investments and repayments the model is:
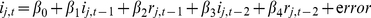
(1)where 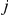 indexes the subject and 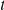 indexes the current round of the game. For a second order dependence of the current proposer investment on previous investments and repayments, this expression would accrue all possible second order terms in lagged investments and repayments, including terms of the type 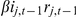 that describe interaction between investments and repayments. Such terms acknowledge that the current choice by the investor 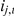 is entangled with their previous interactions with their partner. Although expression 1 depicts a first order dependence on previous investments and repayments extending back two rounds of the game, in this paper, we do not pre-commit to the exact functional dependence for the current proposer investment nor to the number of exchanges into the past that best predict the person's current decision. Instead, we assume a general polynomial dependence of the current investment ratio 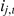 on previous investments and repayments, and determine the order of this polynomial dependence directly from the data. Similarly, we determine the number of rounds into the past required to predict optimally the person's current investment ratio from previous investment and repayment ratios. The details of this general approach follow.

Formally, we model the behavior as a mixture of regressions. For a fixed order of polynomial dependence 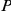, a fixed look-back window 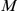, and a fixed number of clusters 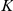, we assume a single investor's data is given by
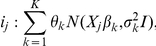
Here, 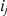 is an investor's (8-dimensional) vector of investments (we consider models looking back as many as two rounds; to make the models comparable we only consider 8 investments), 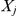 is the model matrix of independent variables defining the regression (all less than or equal to 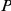-degree monomials in lagged investments and repayments going back 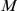 rounds), 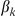 is the 
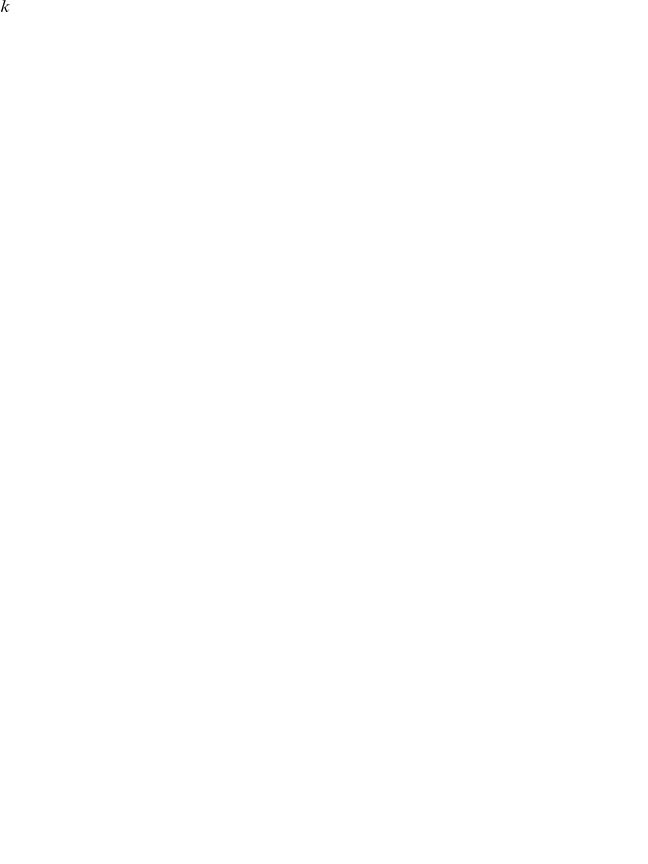
-th cluster's regression coefficients, 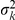 is the variance of the error term in the 
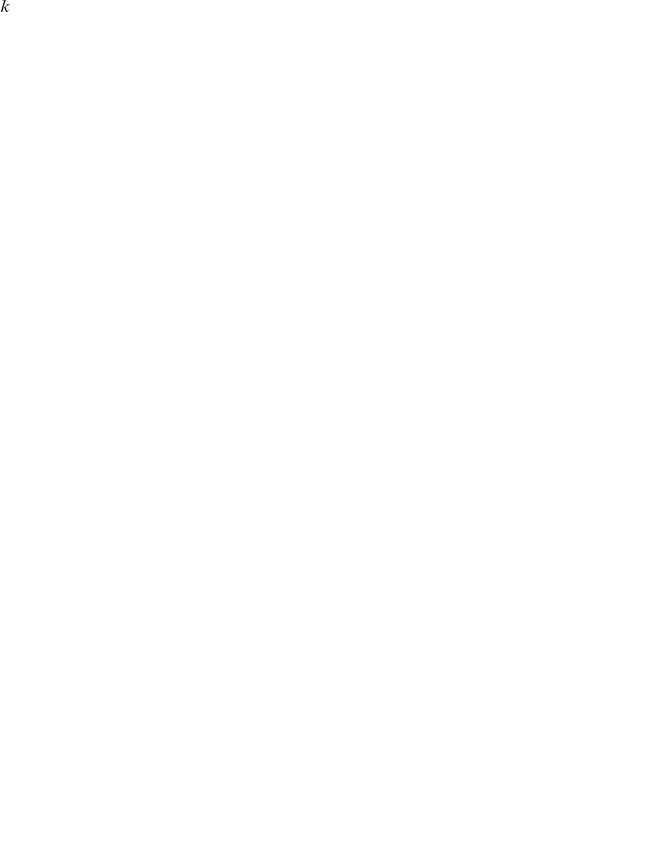
-th cluster, 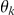 is the weight assigned to the 
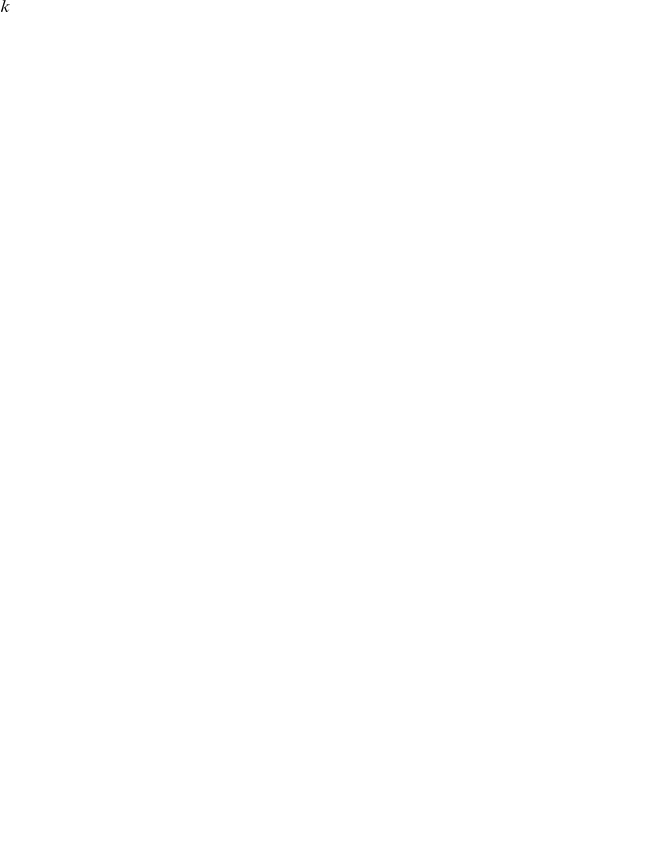
-th cluster, 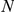 the multivariate normal, and 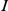 is the identity matrix.

This behavior model is applied to the data from the whole group, with the data itself determining both the appropriate subdivision into clusters and the regression coefficients within each cluster.

We use the data augmentation approach [25], defining latent variables 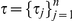 which assign investors to clusters, to form the complete data 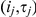. We then get the joint posterior of the parameters and the latent variables by combining the complete data likelihood with priors over the parameters. We choose for priors
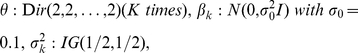
where Dir denotes the Dirichlet distribution and 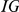 the inverse gamma distribution [26]. These are the same priors that were used by Houser-Keane-McCabe in their work [23]. As our independent variables all lie on the interval 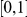, we chose the prior variance of the coefficients to be proportional to this range.

### Estimating the parameter

For the above model, we use a two-stage Gibbs sampling algorithm to estimate the parameters [27]:

Start with initial parameters 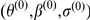 then repeat:

Step 1: Sample allocations 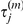 given 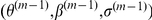: 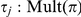, where Mult is a multinomial distribution, and 
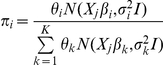
.

Step 2: Sample 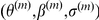 given 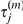
's:
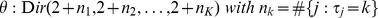
for 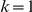 to 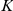


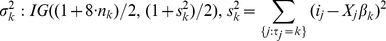


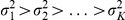


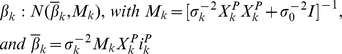
Here, 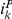 is the pooled investment data over cluster 
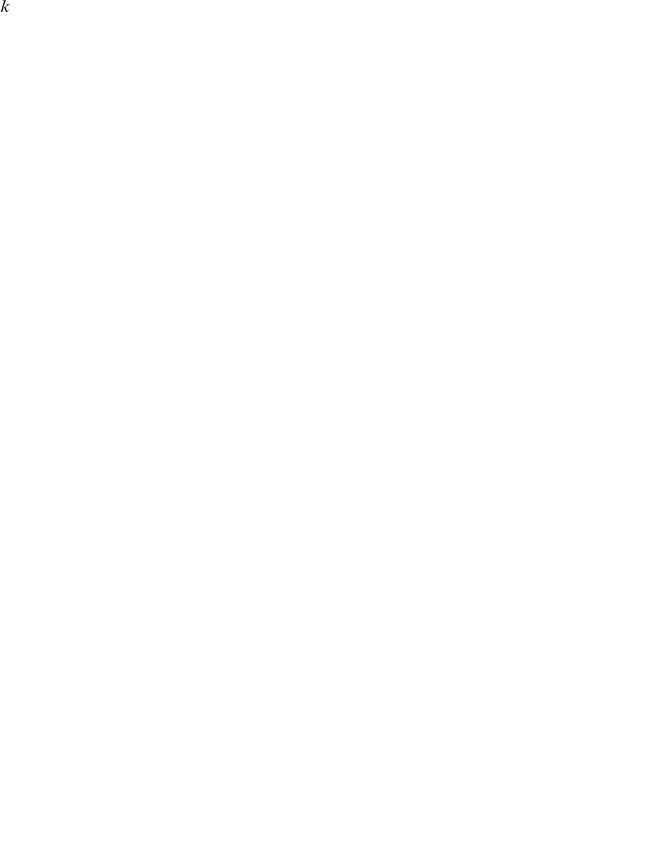
, 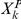 is the pooled model matrix over cluster 
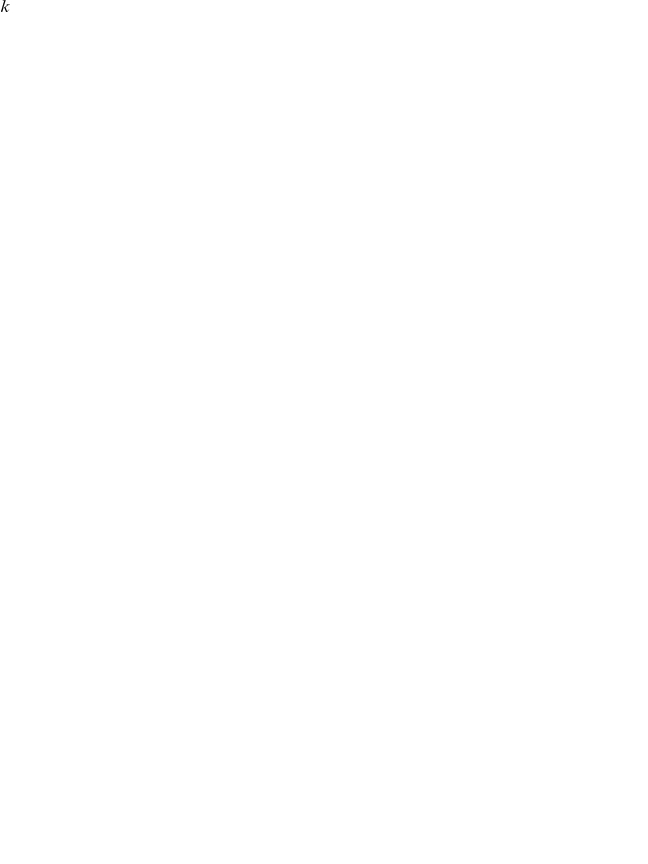
, and 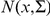 is the normal multivariate density with mean 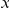 and covariance 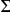. The sequences of samples can then be used to estimate parameters. To avoid possible adverse effect of potential outliers on this Gaussian-based (hence outlier-sensitive) method, we check that the empirical distribution of the differences 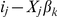 between the observed and predicted values is indeed consistent with the normality hypothesis. Finally, the optimal number of clusters, polynomial order, and look-back window can be determined by computing the marginal likelihood of each model (see the Methods section for details) and selecting the model with the largest value.

### Mapping the Bayesian classification of healthy proposer behavior onto DSM phenomenology of responder

The method described above identified 4 clusters. In terms of the relevant parameters, two rounds were found to be the optimal number of previous moves for predicting the influence of past investments and repayment ratios on the current investment ratio made by the investor. To connect our clusters to the DSM-IV phenomenology, we determined which groups of subjects defined by DSM-specific criteria were over- or under-represented in each cluster and the number of standard deviations by which they were over- or under-represented.

**Figure 2:**
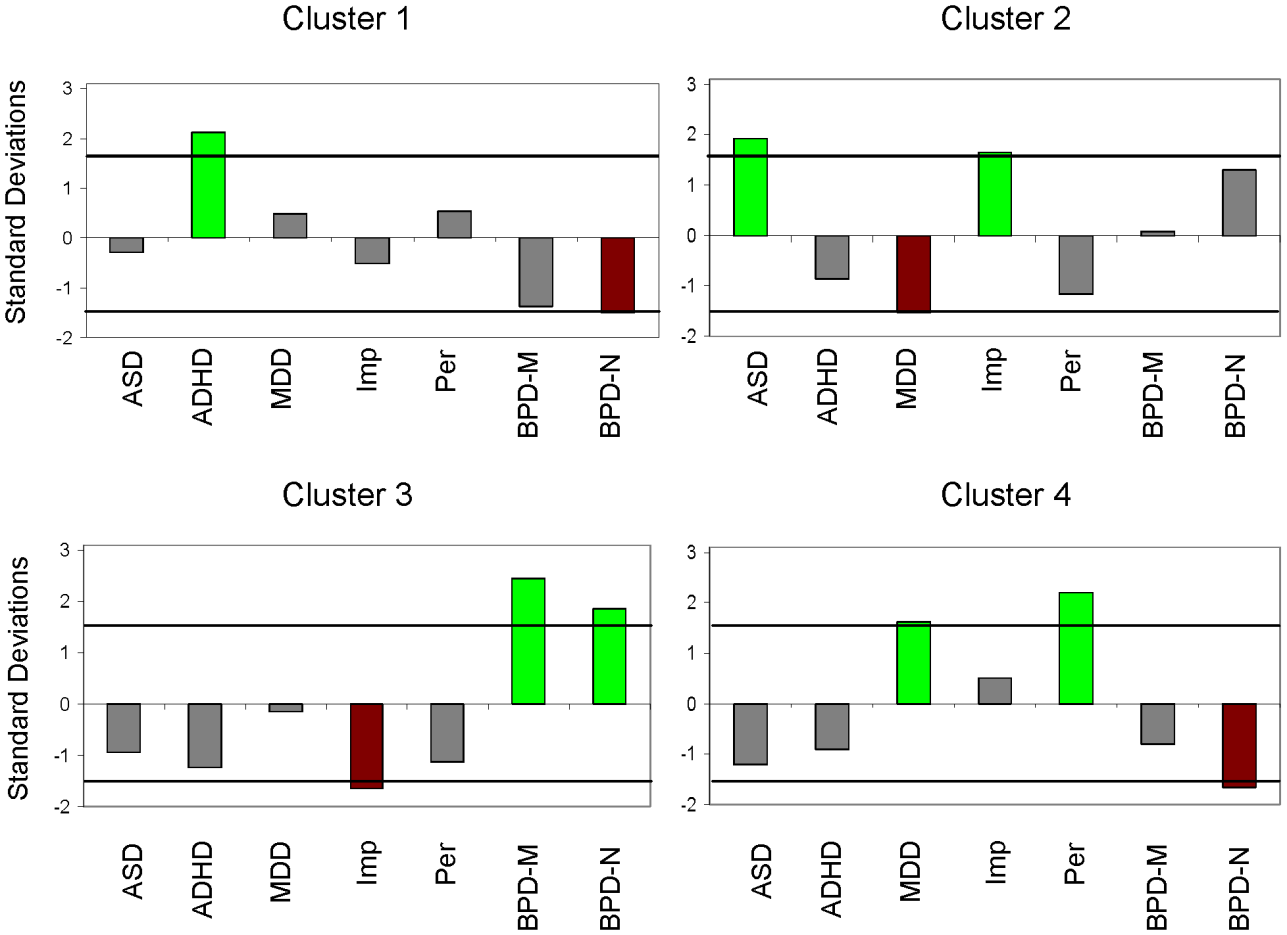
Groups over-/under-represented in behavioral clusters. We analyzed over-/under-representation of original groups in our clusters. Our approach is depicted in Figure 1 and detailed in Materials & Methods section. We used the most frequent value of a dyad's cluster assignment over all draws from the posterior to assign a type for this analysis. We computed the number of standard deviations over-/under-representation in the cluster as compared to that expected by chance. These values are shown for each cluster and each original group. ASD = Adolescents with Autism Spectrum Disorder [20]; ADHD = Children with Attention-Deficit/Hyperactivity Disorder; Per = Healthy individuals who met before playing the trust game [16]; Imp = Healthy individuals who played the trust game remotely with individuals from the California Institute of Technology [7]; BPD-M = Medicated individuals with Borderline Personality Disorder [6]; BPD-N = Non-medicated individuals with Borderline Personality Disorder [6].

The results of the clustering are shown in Figure 2 (see Table S2, Table S3 and Table S4 for a detailed description). *Cluster 1* over-represents individuals with ADHD. Although 54% of these individuals would be expected to fall into this cluster by chance, 89% of them end up in this cluster. *Cluster 2* significantly over-represents individuals with Autism Spectrum Disorder. By chance, 23% of these individuals should fall in this cluster; however, we see 44% of them in the cluster. In *Cluster 3*, medicated and non-medicated individuals with Borderline Personality Disorder are over-represented. By chance, 15% of individuals from each group should fall into this cluster. However, 36% of medicated and 27% of non-medicated Borderline Personality Disorder individuals belong to this cluster. *Cluster 4* should by chance represent 8% of individuals with MDD, but 20% of them fall into this cluster. The chi-square analysis confirms the statistical significance of this over-representation (see Methods section).

### Additional result: probability of belonging to a cluster is correlated with the severity of the disorder

**Figure 3:**
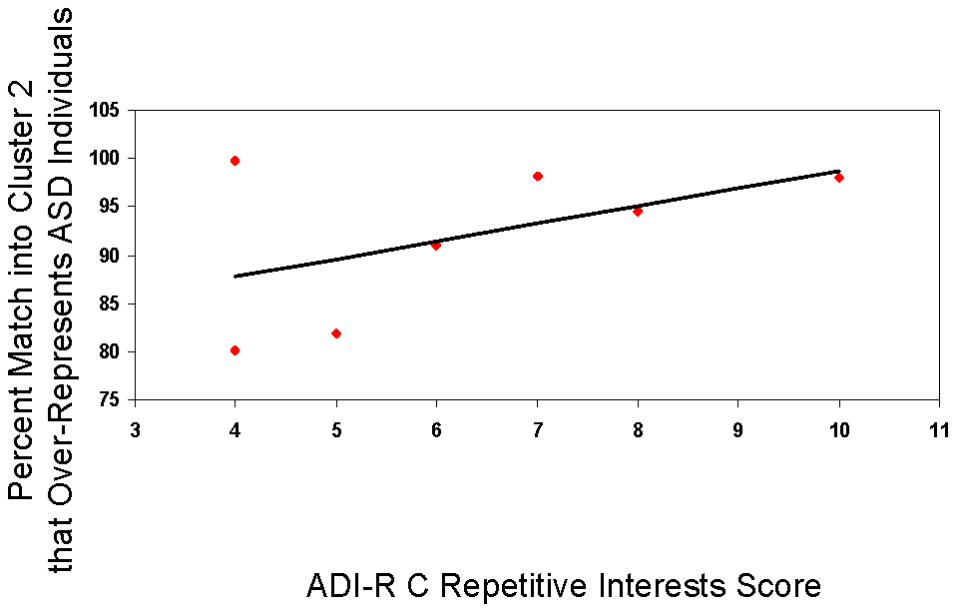
ADI-R C, repetitive interests score, correlates with assignment of dyads with ASD individuals to cluster 2. For dyads with Adolescents with Autism Spectrum Disorder [20] assigned to cluster 2, in which they are over-represented, we analyzed the correlation of the (i) percent match of the dyad into cluster 2 from 30,000 draws from the posterior and (ii) the score on the Autism Diagnostic Interview-Revised [28] Repetitive Behavior subscale of the ASD individual playing in the trustee role. We found a correlation with 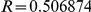 and 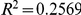.

**Figure 4:**
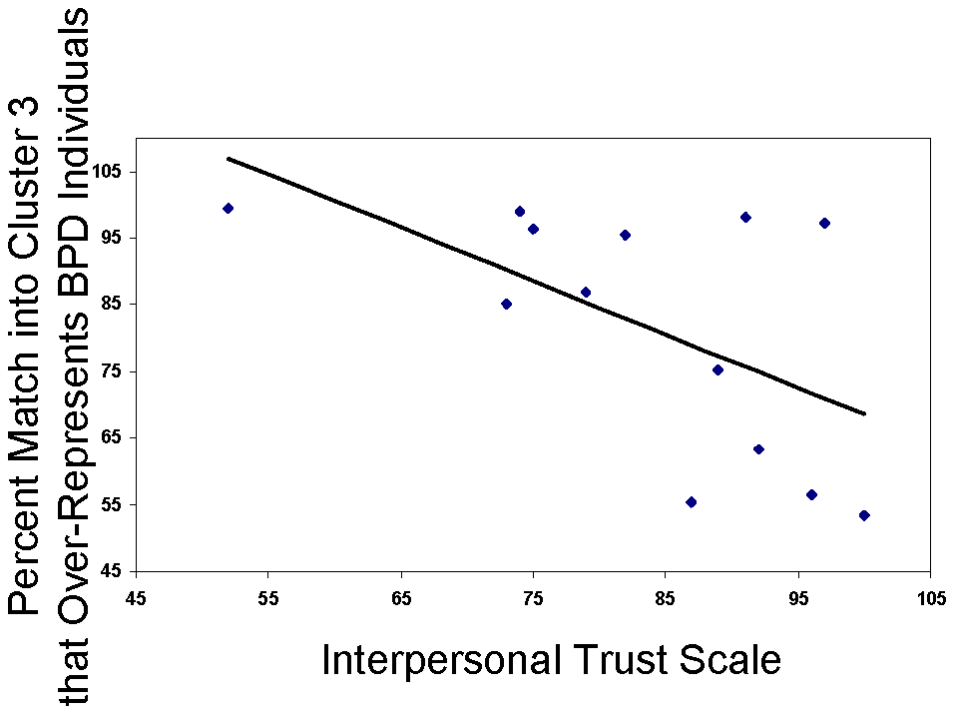
Interpersonal trust scale correlates with assignment of dyads with BPD trustees to cluster 3. For dyads with Borderline Personality Disorder, Medicated and Non-Medicated [6], assigned to cluster 3, in which they are over-represented, we analyzed the correlation of the (i) percent match of the dyad into cluster 3 from 30,000 draws from the posterior and (ii) the score on the Interpersonal Trust Scale [29] of the BPD individual playing in the trustee role (self-report, lower score implies less trust). We found a correlation with 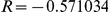 and 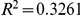 (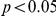).

For two disorders, there are known scores describing its severity: for ASD, there is a score on the Autism Diagnostic Interview-Revised [28] Repetitive behavior subscale, and for BPD, there is a score on the Interpersonal Trust Scale [29]. In both cases, we found a statistically significant correlation between these scores and the probability of belonging to the corresponding cluster ( = percent match of the dyad in this cluster from 30,000 draws from the posterior): 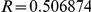 and 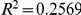 for ASD (Figure 3) and 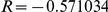 and 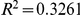 for BPD (Figure 4).

### Characterizing corresponding social behavior

**Figure 5:**
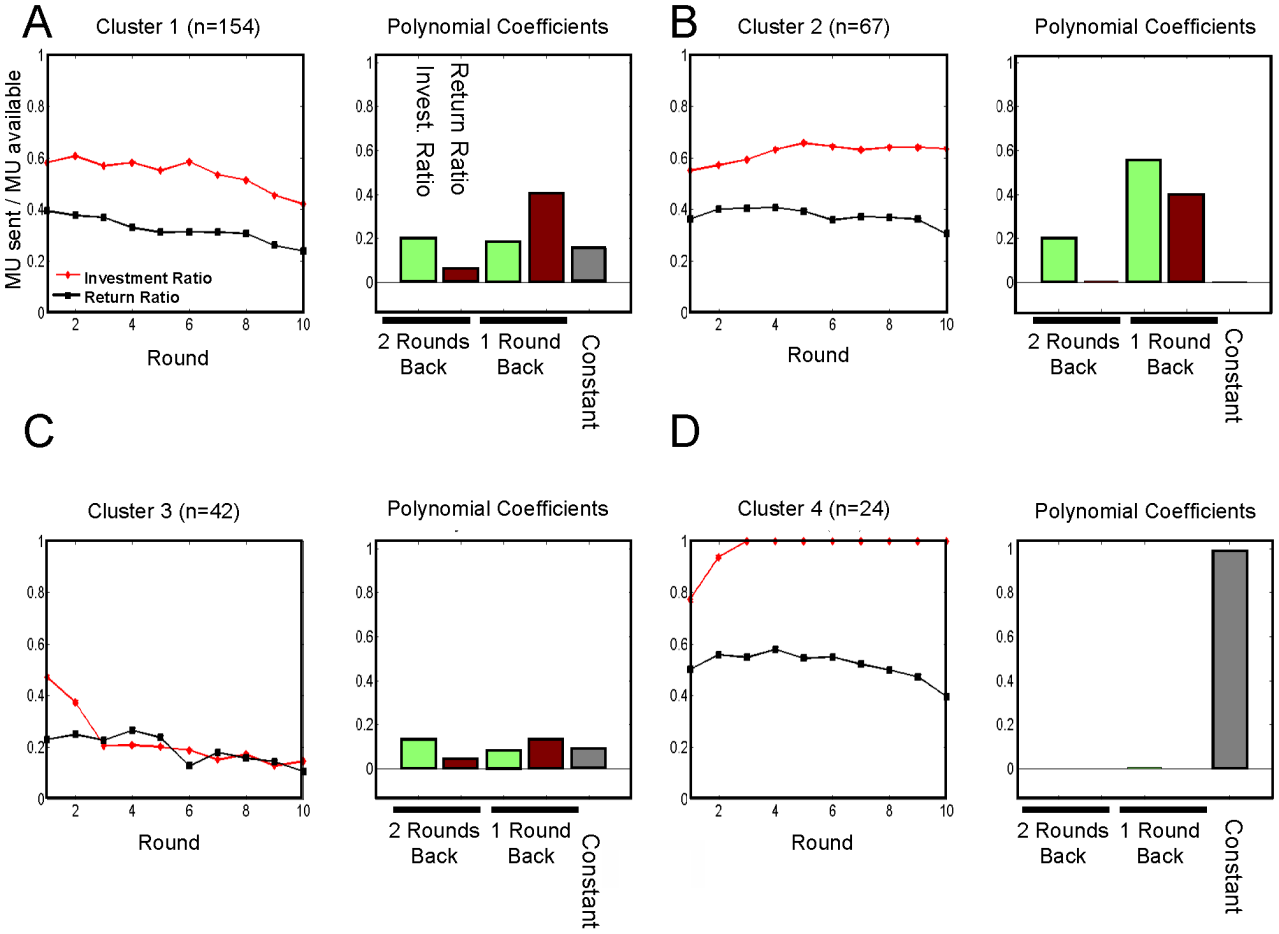
Characteristics of behavioral clusters. A–D) Left Panel: Investor and Trustee Behavior in Behavioral Clusters. For each cluster, the corresponding number of dyads is shown in the title. Further, the corresponding mean investment ratios (red) and return ratios (black) are represented. Standard error of the mean is plotted, but is smaller than the markers used to denote means. Right Panel: Polynomial Coefficients Used to Predict Investment Ratios for Behavioral Clusters. Mean values of polynomial coefficients used to predict investment ratios for each cluster are shown. Specifically, the coefficients by the constant term (gray), return (red), and investment (green) ratios are shown.

With the clusters defined as described, we sought to characterize the kinds of social gestures (signals sent across rounds and between players) that define them. In Figure 5, we summarize the across-round social gestures for each cluster in terms of the regression coefficients for the investment and repayment ratios and the constant term (see also Figure S1 and Figure S2). We discuss the potential importance of these findings below, but here we summarize in Figure 5 the average social gesture of each cluster by plotting the average regression coefficients for each restricting the number of rounds back to two – the optimal number that predicts the investors next investment ratio (Figure 5B). Notice that in Cluster 4, the dependence is dominated by the constant term; this term reflects universally high investments. In Cluster 4, investors playing subjects with major depressive disorder are over-represented. The other over-represented group in Cluster 4 are investors playing trustees that they meet before the game and whose pictures they see each round of the exchange. It is interesting to note that investors playing subjects with ASD end up over-represented in the same cluster (Cluster 2) as investors playing subjects in an impersonal version of the game – where subjects do not meet nor see each other.

### Computer agents as investor-side biosensor

The above results provide evidence that examining investor-side behavior provides a new kind of ‘readout’ for some important psychopathology groups studied under the probe of the multi-round trust game. The game itself, although simple (in each round only two numbers are exchanged), requires a number of intact cognitive functions including working memory, short-term memory, the capacity to model and predict the partner's likely response, the capacity to sense deviations from these expectations, good a priori models of human trade instincts (reflected by round one offers and responses), and so on. One value of this approach is that it utilizes a probe that is not directly related to the symptom lists that define DSM classifications, and therefore provides a possible alternative method of classifying some psychopathologies – or at least identifying or isolating some of their malfunctioning computations.

To verify the robustness of the clustering algorithm we employed a previously described computer agent designed to play the trustee role.

The possibility to design agents of this type was shown in our previous work [6]. The corresponding “
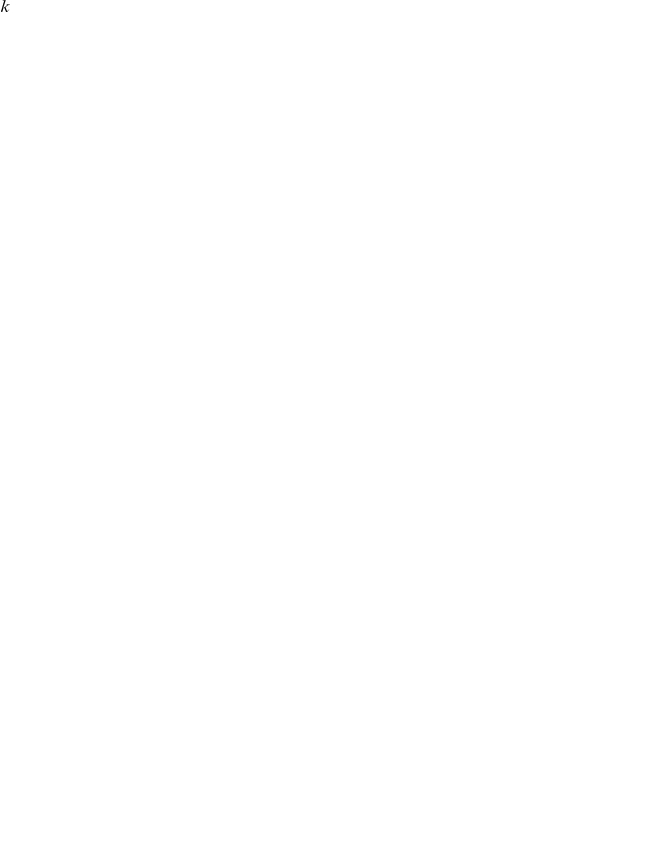
-nearest neighbor” agents use the database containing the results of all the rounds of all the dyads. A *healthy trustee agent*, to describe how much to repay, looks at the vector of 6 previous choices (last 3 investments and last 3 repayments) and finds, of all the records with healthy trustees, 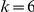 situations in which corresponding previous choices were the closest (in the Euclidean distance). Out of the 6 recorded outcomes of these closest situations, the agent selects one with equal probability. A *BPD trustee agent* similarly selects from dyads with a BPD trustee.

These trustee agents were validated in [6]: in interaction with healthy human investors, the BPD agent was shown to reproduce accurately ruptures in cooperation normally observed when a healthy investor plays a BPD trustee. Such ruptures were not observed in healthy investors playing a healthy computer trustee.

In our case, we need to supplement these agents with a similar *investor agent* that select the investment value based on the 6 closest dyads. Our hypothesis is that the same correlation with disorders can be detected by players playing against the investor agent.

**Figure 6:**
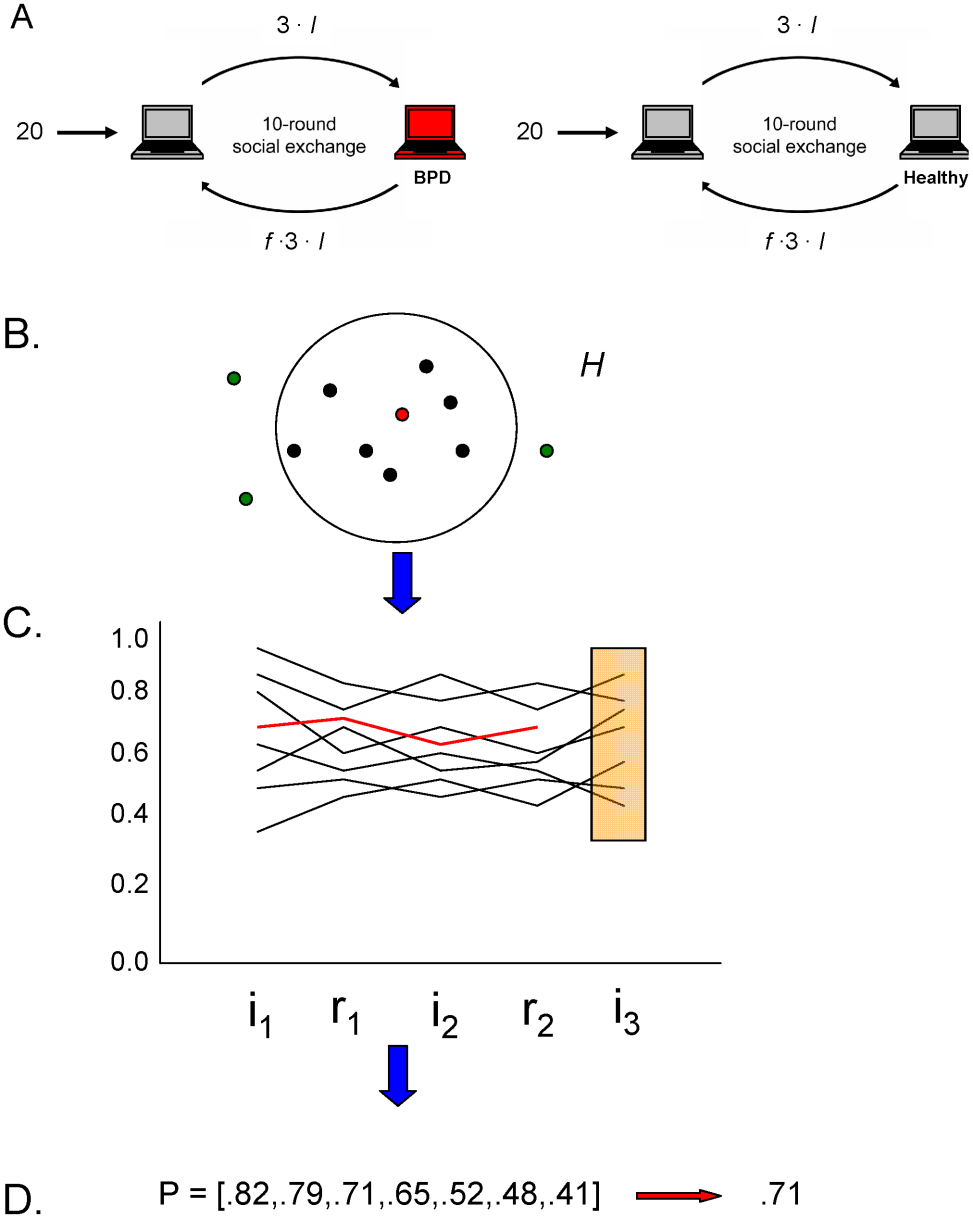
Agent-vs-agent validation of clustering scheme. A) Depiction of agent-vs-agent trust task. Specifically, a k-nearest neighbors agent that samples healthy investor behavior plays the multi-round trust game against a 
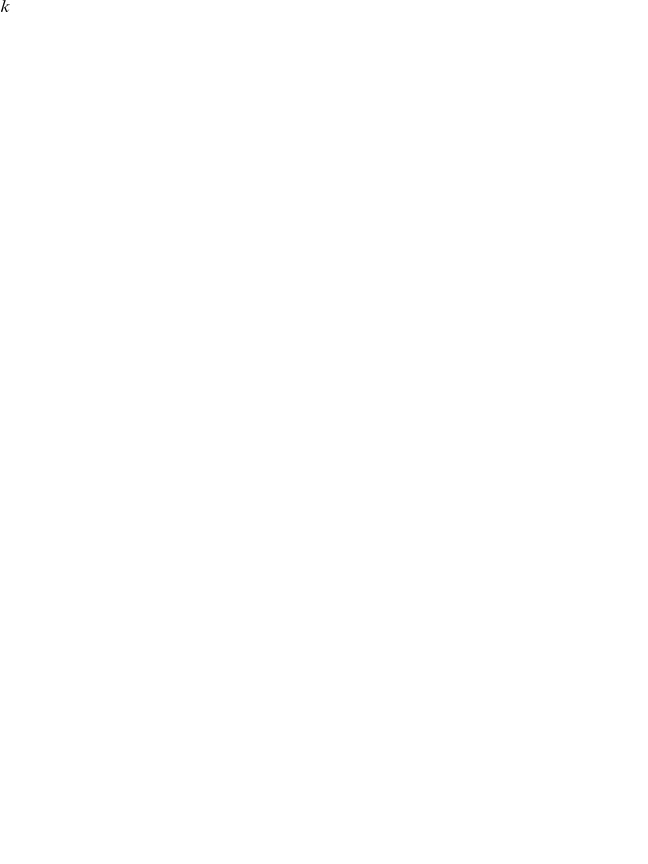
-nearest neighbors agent that samples healthy or BPD trustee behavior for ten rounds [6]. B) Depiction of the space of sampled interactions. The sampling agent uses the records of investment and return from the trust game as played by either (i) healthy trustees, (ii) healthy investors, or (iii) BPD trustees depending on the specific agent used. The agent starts with a vector representing several immediate past choices for the game that is currently playing (this vector forms the center of the circle), and selects several records for which the corresponding vectors have the smallest Euclidean distance to the current vector (these vectors are inside the circle). C) The sampling agent finds the next investment (or return) ratios for all the closest recorded game trajectories. In Panel C, these ratios represent 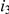. D) The agent then selects, with equal probability, one of these “next” ratios and returns it as the investment (or return) ratio for the game that is currently playing.

**Figure 7:**
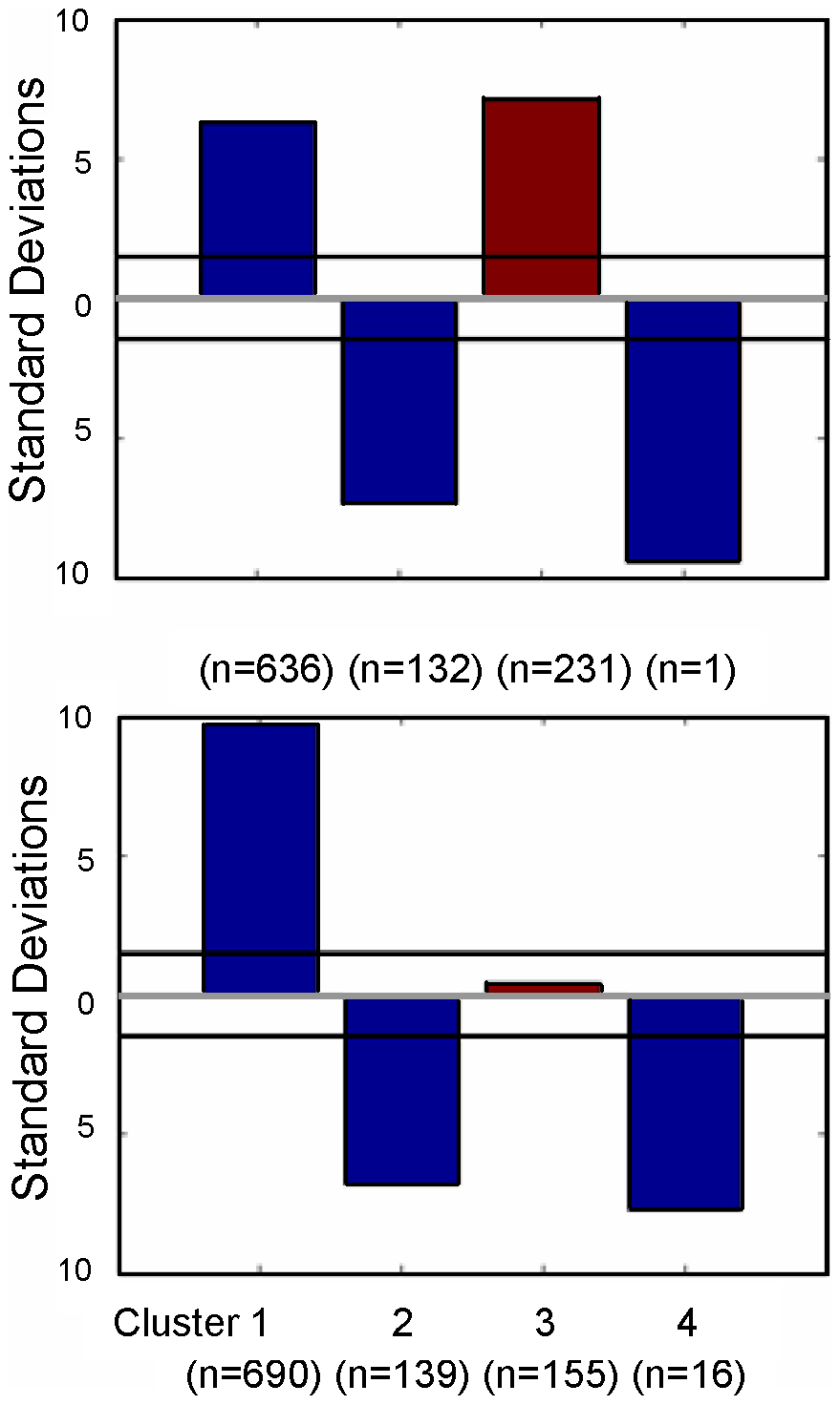
Simulated data over-/under-represented in behavioral clusters. The analysis detailed in Figure 2 was repeated with the Simulated Interactions. In Figure 2, Cluster 3 over-represents healthy individuals playing BPD trustees. Similarly, we compared the number of standard deviations by which in our analysis of simulated interactions, Cluster 3 over-represents simulated healthy-vs-BPD interactions by 7.19 standard deviations. On the other hand, Cluster 3 over-represents healthy-vs-healthy simulated interactions only by 0.46 standard deviations.

Since it was already shown that the trustee agents adequately describe the trustee behavior, we had healthy investor agent play either the healthy or BPD trustee agent in the trust task for ten rounds (Figure 6A) 1,000 times. These interactions were then assigned to the previously determined clusters using the posterior distribution of parameters generated from the analysis of the human dyads (see details in the Methods section). Notably, interactions between the BPD trustee and healthy investor agent were statistically significantly over-represented by 7.19 standard deviations in Cluster 3 – the same cluster in which investors playing both medicated and non-medicated individuals with Borderline Personality Disorder are over-represented. On the other hand, interactions between the healthy investor and healthy trustee agents were not statistically significantly over-represented in this same cluster; see Figure 7 and Figure S3.

Thus, for BPD, the same correlation between the statistical clustering and disorders can indeed be achieved by using the investor agents (For the ASD group, there were insufficient data (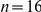) to develop an analogous trustee agent and so no validation along this psychopathology was possible at this time).

## Discussion

We have used a data-driven, Bayesian regression approach to cluster the healthy investor behavioral data from a large set of 287 trust interactions, which included trustees from several DSM mental-illness groups. The Bayesian approach allowed us to determine in a principled way the number of clusters in our population (four) and probabilities for each dyad to belong to each cluster. Next, we used a chi-square criterion for over/under-representation to determine which pre-defined DSM-IV groups are statistically significantly over- and under-represented in each cluster. We found that there is a one-to-one correspondence between the resulting clusters and the DSM-IV disorders: namely, dyads in which trustees have a certain DSM-IV disorder are over-representedin the corresponding cluster.

Moreover, there is a correlation between the severity of each disorder and the probability of belonging to the correpsonding cluster.

The finding that a trustee's disorder can be detected based on the investor's behavior is in line with the fact that in any multi-round interaction with another human, a player's choices are dramatically entangled with those of her partner. Humans bring highly developed social sensitivities to two-party interactions. Our results show that these sensitivities can serve as a *biosensor* – the quantitative behavioral dynamics of a healhy person can capture the subtle behavioral abnormalities (abnormalities that are difficult to capture by the usual statistical analysis) of her partner.

To further validate our approach, we used a previously described 
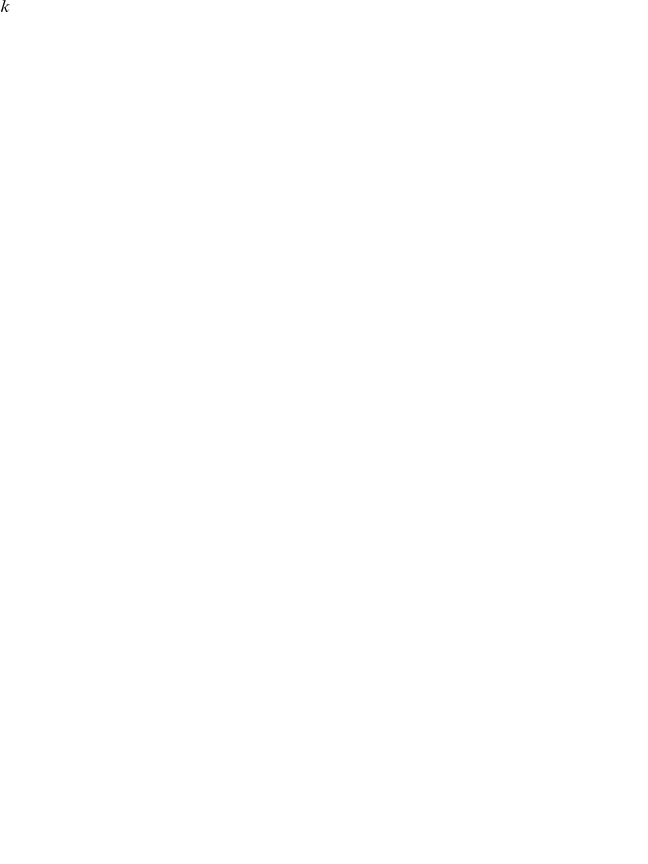
-nearest neighbor sampling agent, as well as its implementation in the investor role, to simulate healthy vs healthy and healthy vs BPD interactions. We showed that healthy vs. BPD agent interactions were over-represented in the same cluster as healthy vs BPD individuals, whereas the healthy vs. healthy agent interactions were under-represented in this cluster.

Having arrived at an initial validation of our clustering, one can ask what further information can we extract from our method. Specifically, what do the patterns of play (Figure 5A) and polynomial coefficients used to predict investor behavior (Figure 5B) tell us about the behavior of individuals in each group?

We start with the fourth, and smallest, cluster. This cluster over-represents (i) dyads who met before playing the trust game as well as (ii) healthy investors playing trustees with Major Depressive Disorder. In this cluster, investment ratios are very high, and return ratios, in comparison to other clusters, are also high. For this “trust cluster”, the constant term effectively dominates the polynomial predicting the investment ratio.

The third, next largest, cluster, over-represents both medicated and non-medicated individuals with Borderline Personality Disorder. In this cluster, both investment and return ratios are relatively low.

The second cluster over-represents adolescents with Autism-Spectrum Disorder. The difference in pattern of play between this cluster and cluster one is difficult to detect by simply looking at the round by round average investment and repayment levels. Notably, the two clusters separate individuals with Autism-Spectrum Disorder from individuals with ADHD, two disorders that are often difficult to separate because they share several symptoms. One of the advantages of our method is that we arrive not only at clusters, but also at polynomial coefficients that can be used to predict investment ratios in each cluster. By looking at these coefficients, one can see a characterizing feature of cluster two - specifically, the current investment ratio depends strongly on the ratios of investment and return one round back. It is known that reciprocity is a driving signal in the trust game [25], and that the sensitivity to reciprocity of individuals with Autism-Spectrum Disorder is blunted [28]. The investor behavior in cluster may be an adaptation to this diminished sensitivity.

While our results show the statistically significant biosensing of certain disorders, the resulting clustering does not provide us with a clear diagnostics – since each cluster contains, in addition to individuals with the corresponding disorder, also a large number of healthy individuals; see Table S5. The fact that we did not get a clear separation between normal participants and participants with disorders (i.e. we find healthy participants scattered across the cluster) points to two distinctly different ways to approach psychopathology [30]. One possibility is that psychopathology groups are reflections of “quantitative” differences along normal cognitive dimensions (and their correlations) that are probed by our interpersonal exchange game. The second is that the first possibility holds but is augmented by the fact that psychopathology groups bring extra (or fewer) or different cognitive dimensions to the responses elicited by the game (Figure S4). To shed light on this issue in the context of this task, we clustered the healthy dyads alone and then assigned the disordered dyads to these clusters. The algorithm again selected 4 as the optimal number of clusters, 1 as the polynomial order, and 2 as the lookback length, but the assignment of the disordered dyads to the clusters is somewhat different than in the main result (Table S6). For BPD dyads the overrepresentation result is stronger, but for the other groups it is weaker. Also, while the betas of the regression (Table S7) are quite similar in three of the clusters, the fourth (Figure S5) is substantially different. Finally, the cluster assignments of the healthy dyads are in good concordance across the two clusterings (adjusted Rand index of .94 [31]–[33]; see also Table S8). Taken together, these facts suggest that the second view of psychopathology mentioned above is to be favored in this task, and that as far as this behavioral probe is concerned the disordered individuals are qualitatively different.

Interestingly, a seemingly more direct classification – based directly on the return values – does not lead to such a statistically significant correlation between clusters and disorders: many differences between healthy and pathological trustees cannot be detected against the background of other behavioral differences; see Table S9. This shows that humans acting as biosensors have the ability to “filter out” the important differences – and thus, help in diagnosing psychopathologies.

To summarize: we have data from 287 dyads involved in one such task - the trust game. We use a data-driven, agnostic method [22], [23] to arrive at (i) the number of clusters, (ii) the order of the polynomial that predicts investment ratios, and (iii) the number of rounds prior on which investor decisions depend directly from the data. We then arrived at a probabilistic clustering of these dyads, and analyzed over-representation of initial groups in the new clusters. We found that, by clustering dyads based on investor decisions, we were able to over-represent trustees with different disorders in separate clusters. Further, we used previously described k-nearest neighbor sampling agents [6] to generate 1000 interactions each for healthy vs healthy and healthy vs BPD agents. By clustering these interactions based on the polynomial coefficients from our initial clusters, we found that simulated healthy vs BPD interactions are statistically significantly over-represented in the same cluster as real healthy vs BPD interactions, but that simulated healthy vs healthy interactions are statistically significantly under-represented in the same cluster. We believe that these results constitute a significant step forward in quantitative diagnosis of psychiatric illness. The fact that brain images have helped in the analysis of human behavior in fairness games [2]–[8] makes us believe that our diagnoses can be further refined by using the corresponding brain imaging data.

Current psychiatric diagnoses are based on the DSM [34]. Essentially, these are lists of criteria used by a trained physician to characterize whether or not a person has a specific disorder. Such clinical, experience-based classification schemes provide a valuable understanding of psychiatric and neurological disorders. However, to uncover genetic underpinnings of various psychiatric disorders and to provide quantitative behavioral and neural measures, it is desirable to have quantitative measures of normal social interactions that can expose computations perturbed in various psychopathologies. Such measures could then be used to quantify abnormalities in social exchange, to diagnose psychiatric and neurological disorders, and to probe the genetic basis of such disorders. The results presented in this paper show some of our first steps in this direction; however, as more data on this and similar parametric social exchange tasks becomes available it should help to construct a quantitative understanding of mental disorders.

### Additional observations

Intuitively, one might expect the investment on the next round to be an interactive function of both previous investment and the repayment the investor received, rather than independent effects of each. However, our analysis shows that the optimal clustering corresponds to polynomials of order 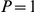, i.e., to the linear dependence (1). This means that, contrary to this intuition, the second-order terms – in particular, interaction terms between investments and repayments (such as 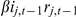) – do not lead to a statistically significant improvement of the model's explanatory power.

For patients diagnosed with a DSM-IV disorder, medication is an important potential confound. In our study, only some BPD patients were medicated. According to Figure 2, both medicated and non-medicated BPD patients were statistically significantly over-represented in the corresponding Cluster 3. Thus, the presence or absence of medication does not affect our classification.

In this paper, we use a purely data-driven approach to data analysis. This approach is important from the foundational viewpoint, since it enables us, in particular, to further confirm the objective nature of the existing psychopathology classification. From the practical viewpoint, once this classification is established, we can improve the diagnostic efficiency if we explicitly use the known diagnoses in classification and regression analysis. For example, this may make it possible to find the markers that identify healthy subjects with superior discriminatory power.

## Materials and Methods

### Ethics statement

Informed consent was obtained for all research involving human participants, and all clinical investigation were conducted according to the principles expressed in the Declaration of Helsinki. All procedures were approved by the Baylor College of Medicine Institutional Review Board.

### Multi-round trust game

The game is described in the previous section. Healthy participants were invited to the Human Neuroimaging Laboratory at Baylor College of Medicine. Prior to playing the game, each participant was instructed they would earn between $20 and $40, scaled by number of monetary units (MU) each player individually accrued. Following the game, each participant was compensated as follows: <68 MU = $20, 68–133 MU = $25, 134–200 MU = $30, 201–300 MU = $35, and >300 MU = $40.

### Bayesian classification: Gibbs sampler

We discarded 1,000 draws as burn-in, sampled 30,000 draws from the posterior, and assessed convergence using the Raftery-Lewis test [35]. We used the R Bayesian Output Analysis program to perform these calculations [36]. We repeated our analyses using 8,000 cycles total as per Houser, Keane, and McCabe [23] and 1,000; 3,000; and 5,000 cycles as burn-in and arrived at similar over-representation results.

### Checking normality

To check that the empirical distribution of the differences 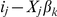 between the observed and predicted values is indeed consistent with the normality hypothesis, we normalize each difference by subtracting the sample mean of the differences from the corresponding cluster and then divide by the sample standard deviation of these differences. We then compute the sample skewness and the sample kurtosis of the collection of all these differences, and use Matlab's Jarque-Berra test to check normality. Normality has been confirmed with 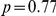. Since the null hypothesis of normality is rejected when 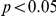, our value of 
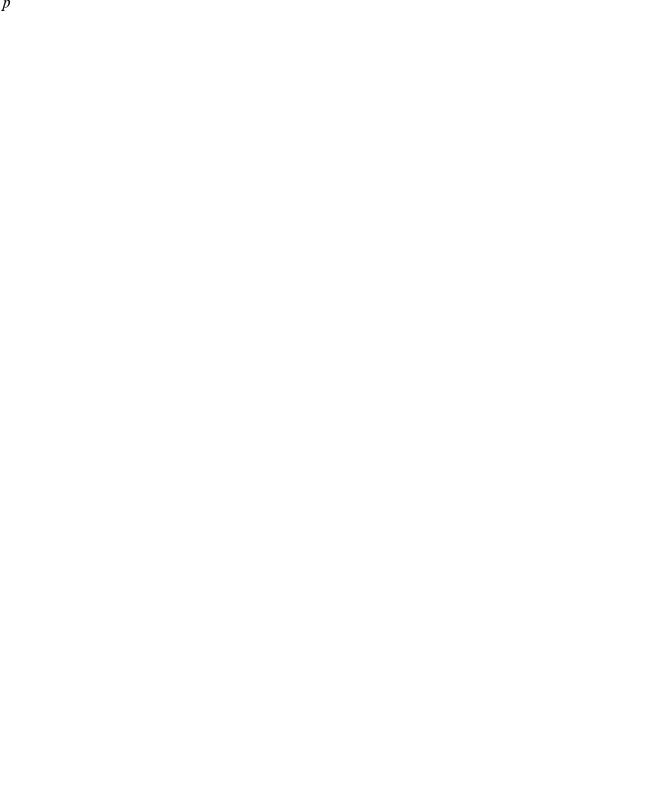
 indicates a strong empirical support for the normality hypothesis.

### Bayesian classification: Optimal parameters

We used the Laplace-Metropolis estimator of the marginal likelihood [37], as described in Houser, Keane, and McCabe [23], to compare models with different values of the number K of clusters, order P of the polynomials, and the number D of past rounds on which the model depends. We did not include any results in which 2 of 3 samplers arrived at at least one empty type in the mode of the last 5,000 of 8,000 draws from the posterior. To maximize marginal likelihood (i.e., to find a posterior mode), we used component-wise optimization (also known as conditional maximization or step-wise ascent; see, e.g., p. 312 of [38]), the use of which is well-established for Bayesian problems such as maximizing the posterior mode, and arrived at the same answer when comparing the maximum log marginal likelihoods for different models. As a result, we concluded that the optimal model has 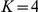 clusters, a first order polynomial 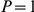, and a dependence of ratios of investment on ratios of investment and return 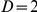 rounds into the past.

We found that, in contrast to the simpler case described in [23], our marginal likelihood values are sometimes fairly close to one another in many cases and thus, the results of comparing these values can potentially change if we repeat the same computational experiment. To makes sure that our selection of 4 clusters does not change, we supplemented the conditional maximization by the exhaustive analysis of all possible triples 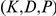 with up to 10 clusters, polynomials of order 1 to 3, and a time dependence of 1 or 2 rounds into the past. For each such model, we used several samplers and got several values of marginal likelihoods; when we compare two models, we select the simpler one (the one with fewest overall parameters) unless the other one has a statistically significantly larger mean. Since for the same model 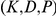, the distribution of marginal likelihood values is sometimes not Gaussian (see Figure S6B), we could not use the usual t-test. Instead, we used the Wilcoxon rank-sum test at the 5% significance level [39]. The results (shown on Figure S6A and detailed in Table S4) confirm that the model with 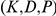 = (4, 1, 2) is optimal.

### Over-representation analysis

To check whether the observed over-representation of participants with disorders in different clusters is statistically significant, we apply the chi square test corresponding to a null hypothesis that the participants of different disorders 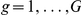 are randomly distributed in different clusters 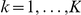.

Let 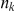 be the number of elements in the 
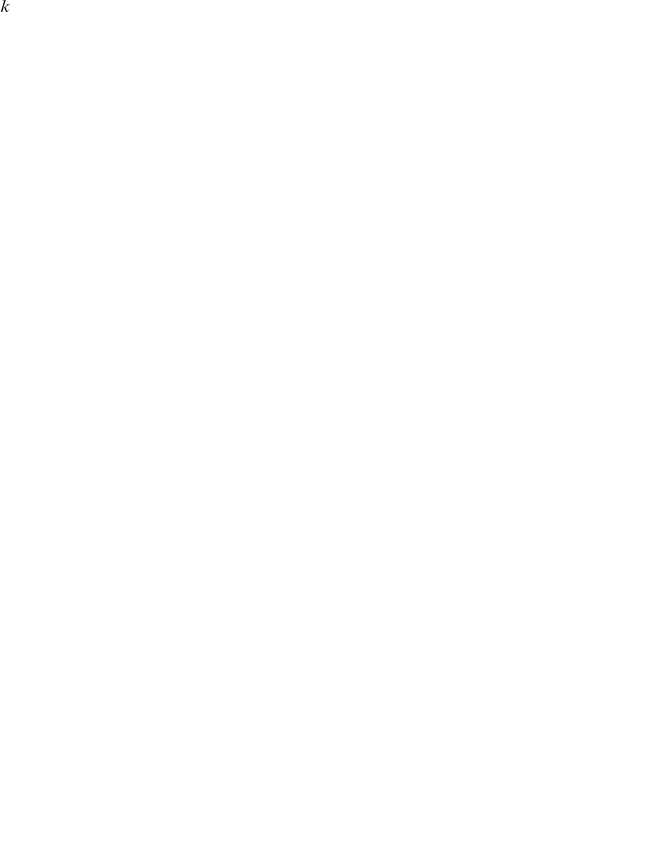
-th cluster, 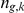 the number of elements of 
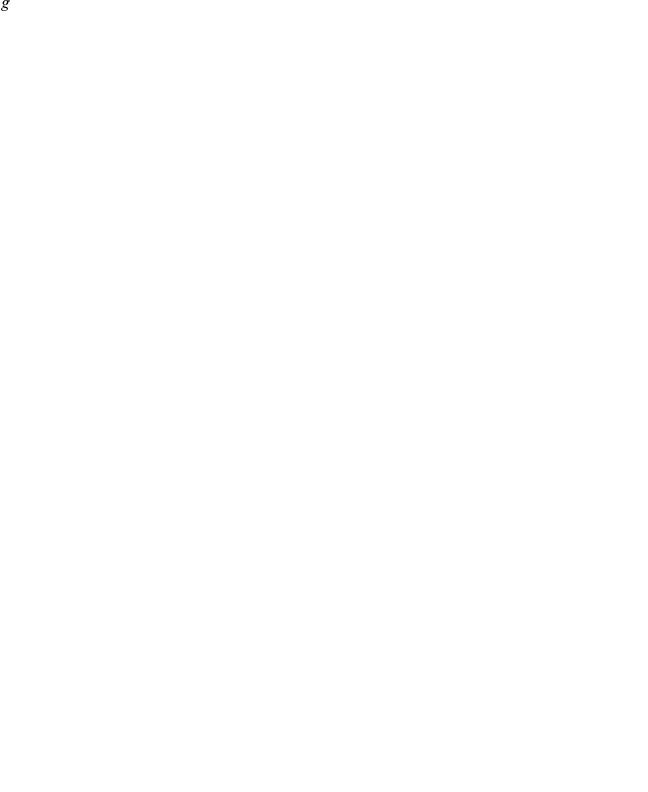
-th group in this cluster, 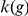 the cluster corresponding to the group 
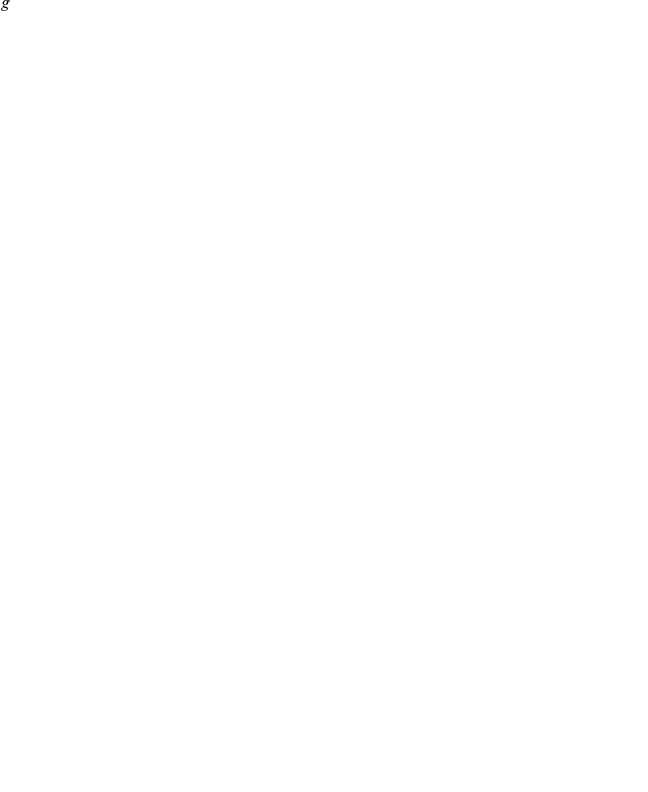
, and 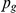 the ratio of group 
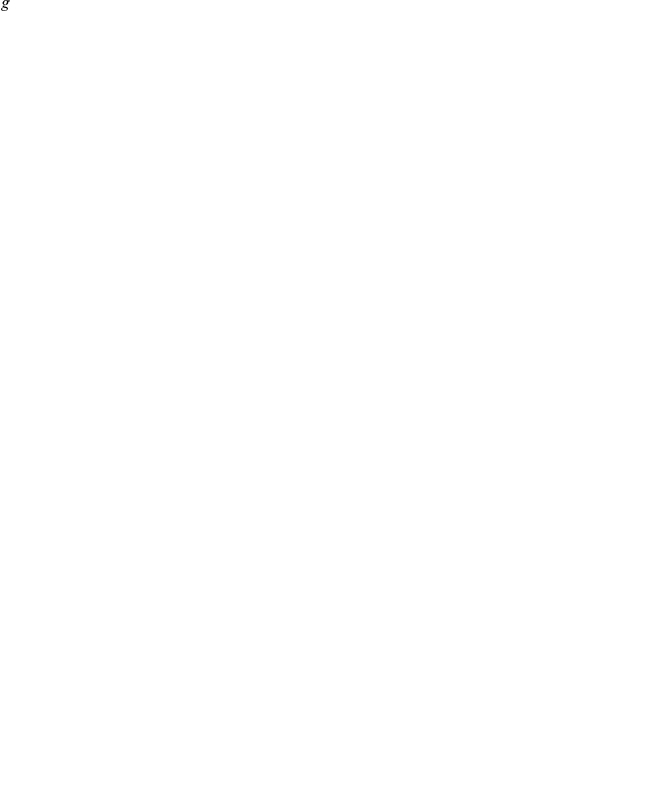
 in the population as a whole. Under the null hypothesis, due to the central limit theorem, the value 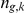 is asymptotically normally distributed, with mean 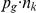 and variance 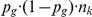. Thus, the ratio 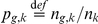 is normally distributed with mean 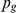 and variance 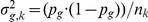. Thus, to test the null hypothesis, we can form the test statistic 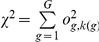, where 
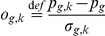
 is a relative over-(under-) representation of the group 
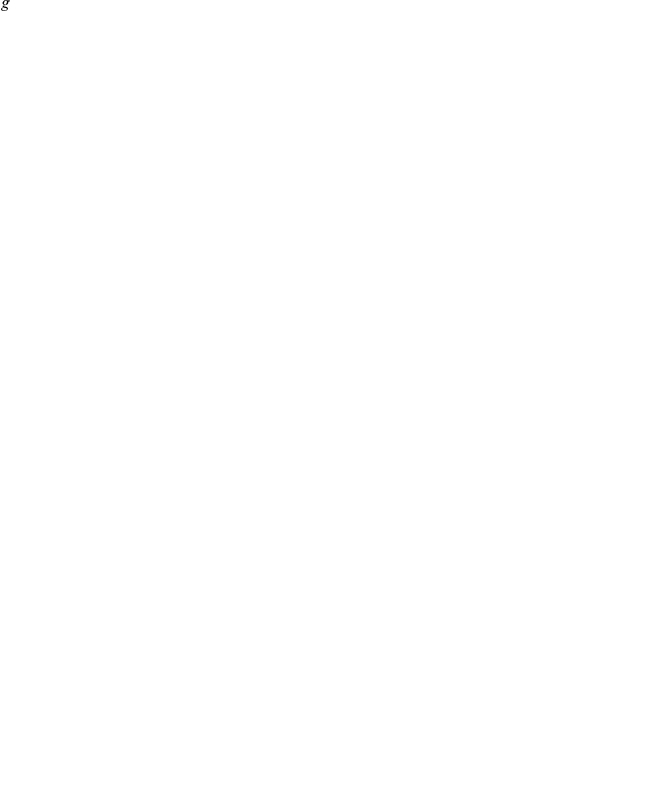
 in the cluster 
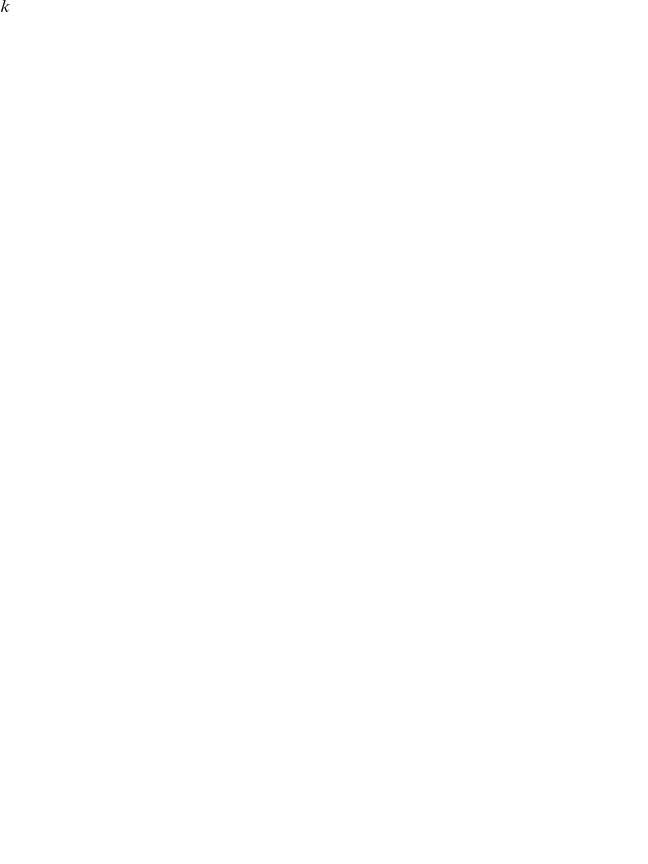
. For 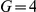, the null hypothesis is rejected with 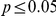 when 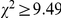. Thus, when each of the four terms 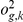 in the sum satisfies the inequality 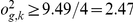, the null-hypothesis is rejected. We therefore consider the groups which are over-represented at the level 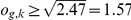.

Please note that when 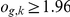, already the over-representation of the group 
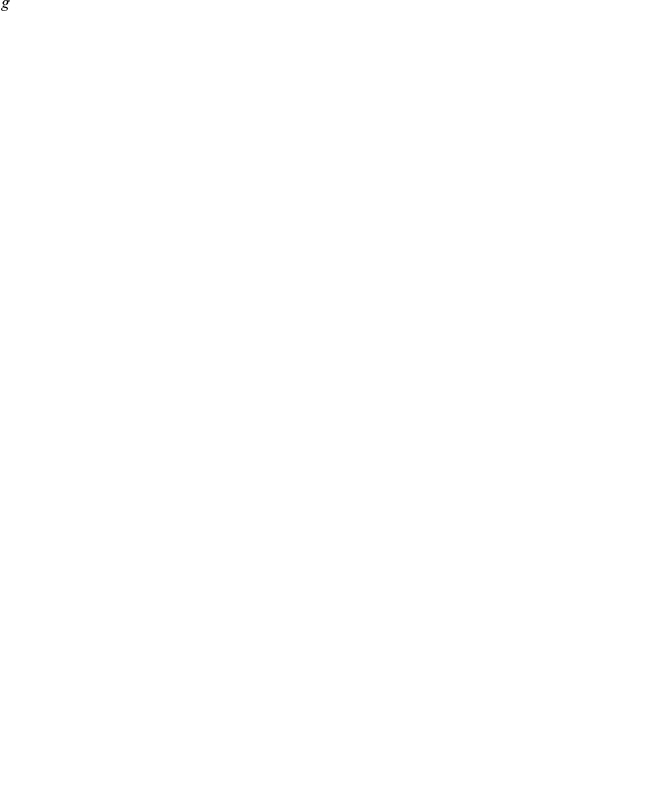
 in cluster 
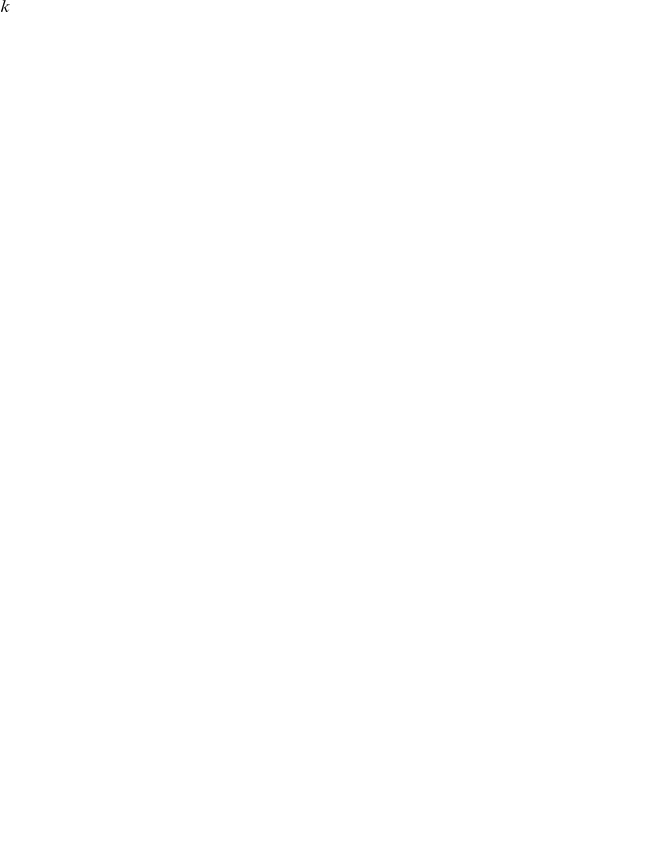
 is statistically significant with 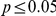.

### Applying resulting classification to other records

Our clustering is based on iterations of Gibbs sampling. Every additional vector 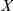 (e.g., of agents playing) is then classified as follows. For each recorded iteration of the Gibbs sampling (after the burn-in), based on the recorded values of 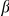 and 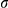, we compute the probabilities 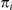 of 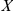 belonging to different clusters 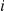 (we use the same formula as in the subsection “Estimating the parameter”). Then, we select a cluster 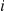 with the probability 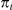. After all these selections, we assign the dyad characterized by the vector 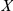 to the cluster to which, among all the iterations, this vector was assigned the largest number of times.

## Supporting Information

Figure S1 Mean ratios of investment and return for all dyads considered in analysis. Ratios of investment ( = MU/20) and return ( = MU/[3*investment amount]) are shown for each of the initial groups considered in the analysis. The number of pairs in each group is indicated in the title. Standard errors of the mean are displayed.
(0.11 MB PDF)

Figure S2 Polynomial coefficient distributions over 30,000 draws from posterior distribution. The polynomial coefficients that predict investment ratios are stable after 30,000 draws from the posterior distribution and are approximately normally distributed. We show a histogram of each polynomial coefficient whose mean is shown in Fig 5. A red line is placed at the zero position, denoting a monomial that does not contribute to the value of the predicted investment ratio.
(0.15 MB PDF)

Figure S3 Mean ratios of investment for agent vs agent interactions. As seen in human players [6], cooperation fails across rounds when the BPD k-nearest neighbor sampling agent engages in a repeated exchange of trust with the control individual k-nearest neighbor sampling agent. Among 1,000 interactions between control agents (gray) paired with control trustees (gray), investments were large and sustained across early (1 to 5) rounds and late (6 to 10) rounds of the game. However, among 1,000 interactions between control agents (gray) paired with trustee agents sampling from interactions with BPD individuals (red), a decrease in investment level from early to late rounds of the game indicates a failure in cooperation across the iterated exchange. Mean percent invested and SEM are plotted.
(0.03 MB PDF)

Figure S4 Clustering healthy versus healthy plus disordered dyads. Here X's represent healthy dyads, while the read and blue o's and +'s represent disordered dyads (the black and white + and −'s represent the projection onto the healthy dimensions). If the healthy dyads are clustered alone, then there would be two distinct clusters along the first axis. The disordered dyads would be assigned evenly across these clusters, resulting in no overrepresentation. In contrast, if all of the dyads are clustered, there would likely be two clusters, one with the o's overrepresented, and one with the +'s overrepresented.
(0.00 MB PDF)

Figure S5 Means and standard deviations of posterior distributions of parameters in four clusters defined by clustering all dyads and healthy dyads only.
(0.04 MB PDF)

Figure S6 Model selection. A) Plot of the log marginal likelihoods computed using the method of Lewis-Raftery [37] used by Houser-Keane-McCabe [23]. We ran 9 samplers for each choice of the number of clusters K, number of rounds to look back D, and order of the polynomial P describing investment ratios in a given round in terms of ratios of investment and return in prior rounds. We used the standard quantile function in MATLAB R14 SP3 (Natick, MA) to compute 95% quantiles for our marginal likelihoods, which are plotted in this graph. B) Histograms of log marginal likelihood values for two samplers, showing that the distributions of the log marginal likelihood values are not Gaussian; thus, the Wilcoxon rank-sum test for comparison of medians was used [39].
(0.04 MB PDF)

Table S1 Available data. All trust game datasets used in the analysis are included here, along with appropriate references if applicable. The number of dyads in each dataset is shown, along with the abbreviations used in other figures and tables.
(0.03 MB DOC)

Table S2 Matching dyads to clusters. We present the most common and second most common match of all dyads in each original group into each new cluster. As can be seen, assignments are relatively stable over 30,000 draws from the posterior distribution (Fig. S2 shows the polynomial coefficient distributions for the same number of draws from the posterior distribution).
(0.25 MB DOC)

Table S3 Groups over-/under-represented in behavioral clusters. We present the number and percentage of each original group matched into each new cluster. The assignment, as in previous tables, is based on the most common match of each dyad over 30,000 draws from the posterior distribution.
(0.04 MB DOC)

Table S4 Model selection. We present log marginal likelihoods estimated using the method of Lewis-Raftery [37] from 9 samplers for each choice of number of clusters K, look-back rounds D, and order of polynomial P describing the dependence of investment ratios in a given round on investment & return ratios in prior rounds. We sort all models by the number of model parameters, and discard models for which > = 6 have an empty type in the mode of all 5,000 draws from the posterior (after the first 3,000 is discarded as burn-in). We used the Wilcoxon Rank-Sum Test [39] to compare a given model's median log marginal likelihood with that of each model with fewer parameters. We chose the model (red) with the largest marginal likelihood for which we can guarantee that it is better than all parsimonious models. We report, in the right-hand column, for each model, the number of the first model for which we cannot guarantee the marginal likelihood is superior; that is, either (i) the median of this model is lower than the median of the model # in this column or (ii) the Wilcoxon rank-sum test, as implemented in MATLAB R14 SP3 (Natick, MA), does not reject the null hypothesis that the medians of the log marginal likelihoods for the two different models come from the same distribution at a 95% significance level. Please also see Figure S5. For the case of two models: K = 4, D = 2, P = 1 and K = 3, D = 2, P = 1, we performed an analysis using 3 samplers to compare the method of marginal likelihood used above based on the posterior mode [25] with a method, the mean harmonic estimator, that is based on using all draws from the posterior (see [38] for a detailed review of this and other methods of calculating marginal likelihoods and model selection). We found, for the median value of 3 samplers for each model, the log marginal likelihood for the Lewis-Raftery method preferred the K = 4, D = 2, P = 1 model by 36.64 units, whereas the mean harmonic estimator preferred the K = 4, D = 2, P = 1 model by 52.99 units. Thus, the two estimates are in good agreement, further justifying our use of the well-established Lewis-Raftery method.
(0.08 MB DOC)

Table S5 Degree of clustering expressed in terms of clinically relevant indices. For each of the four pathologies, this table describes the values of the standard clinically relevant indices: sensitivity, specificity, and positive and negative predictive values.
(0.02 MB DOC)

Table S6 Clustering based on all the trustees vs. clustering based only on healthy trustees. For each of the four pathologies, this table describes the over-representation of participants with this pathology in the corresponding cluster. According to our computations (see Methods section), only over-estimations of 1.5 and larger are statistically significant.
(0.02 MB DOC)

Table S7 Summary statistics of posterior distributions of regression coefficients.
(0.46 MB DOC)

Table S8 Relation between clusters based on all dyads and clusters based on only healthy trustees. This table shows that for dyads with healthy trustees, there is a concordance between their cluster assignments across the two clusterings: when we cluster all dyads and when we only cluster dyads with healthy trustees. Of all the healthy dyads which were assigned to Cluster 1 in the clustering based on all the dyads, 100% got assigned to Cluster 1 in the healthy-trustees-only clustering. Of all the healthy dyads which were assigned to Cluster 2 in the clustering based on all the dyads, 91.9% got assigned to Cluster 2 in the healthy-trustees-only clustering. Of all the healthy dyads which were assigned to Cluster 3 in the clustering based on all the dyads, 95.0% got assigned to Cluster 3 in the healthy-trustees-only clustering. Of all the healthy dyads which were assigned to Cluster 4 in the clustering based on all the dyads, 89.5% got assigned to Cluster 4 in the healthy-trustees-only clustering.
(0.02 MB DOC)

Table S9 Clustering based on the investment ratios vs. clustering based on the return ratios. For clustering based on the investment ratios, different optimization techniques lead to the same optimal number of clusters K = 4. For clustering based on the investment ratios, different optimization techniques lead to different numbers of clusters: K = 2 and K = 5. For each of the corresponding three clusterings, for each of the four pathologies, we list of largest over-representation and, in parenthesis, the number of the cluster in which this over-representation occurs. A blank slot means that the corresponding pathology is not over-represented in any of the clusters. According to our computations (see Methods section), only over-estimations of 1.5 and larger are statistically significant. For clustering based on the investment ratios, all overrepresentations are statistically significant, and different pathology groups are overrepresented in different clusters - i.e., this clustering provides a statistically significant separation of different pathologies. For clusterings based on the return ratios, not all overrepresentations are statistically significant, and some groups are overrepresented in the same cluster.
(0.02 MB DOC)

## References

[1] Trivers RL (1971). The evolution of reciprocal altruism.. Q Rev Biol.

[2] Rilling JK, Gutman DA, Zeh TR, Pagnoni G, Berns GS (2002). A neural basis for social cooperation.. Neuron.

[3] Sanfey AG, Rilling JK, Aronson JA, Nystrom LE, Cohen JD (2003). The neural basis of economic decision-making in the ultimatum game.. Science.

[4] Rilling JK, Sanfey AG, Aronson JA, Nystrom LE, Cohen JD (2004). The neural correlates of theory of mind within interpersonal interactions.. Neuroimage.

[5] Delgado MR, Frank RH, Phelps EA (2005). Perceptions of moral character modulate the neural systems of reward during the trust game.. Nat Neurosci.

[6] King-Casas B, Sharp C, Lomax-Bream L, Lohrenz T, Fonagy P (2008). The rupture and repair of cooperation in borderline personality disorder.. Science.

[7] King-Casas B, Tomiln D, Anen C, Camerer CF, Quartz SR (2005). Getting to know you: Reputation and trust in a two-person economic exchange.. Science.

[8] Singer T, Seymour B, O'Doherty JP, Stephan KE, Dolan RJ (2006). Empathic neural responses are modulated by the perceived fairness of others.. Nature.

[9] Camerer CF (2003). Behavioral game theory: Experiments in strategic interaction.

[10] Camerer CF, Fehr E (2006). When does “economic man” dominate social behavior?. Science.

[11] Kagel JH, Roth AE (1997). The handbook of experimental economics.

[12] Montague PR, Lohrenz T (2007). To detect and correct: Norm violations and their enforcement.. Neuron.

[13] Axelrod R (1984). The Evolution of Cooperation.

[14] Güth W, Schmittberger R, Schwarze B (1982). An experimental analysis of ultimatum bargaining.. J Econ Behav Organ.

[15] Roth AE, Kagel JH, Roth AE (1995). Bargaining experiments.. The handbook of experimental economics.

[16] Tomlin D, Kayali MA, King-Casas B, Anen C, Camerer CF (2006). Agent-specific responses in the cingulate cortex during economic exchanges.. Science.

[17] Camerer C, Weigelt K (1988). Experimental tests of a sequential equilibrium reputation model.. Econometrica.

[18] Weigelt K, Camerer CF (1988). Reputation and corporate strategy: A review of recent theory and applications.. Strategic Management Journal.

[19] Berg J, Dickhaut J, McCabe K (1995). Trust, reciprocity, and social history.. Games Econ Behav.

[20] Chiu PH, Kayali MA, Kishida KT, Tomlin D, Klinger LG (2008). Self responses along cingulate cortex reveal quantitative neural phenotype for high-functioning autism.. Neuron.

[21] Yoshida W, Dolan RJ, Friston KJ (2008). Game Theory of Mind.. PLoS Comput Biol.

[22] Houser D, Bechara A, Keane M, McCabe K, Smith V (2005). Identifying individual differences: An algorithm with application to phineas gage.. Games Econ Behav.

[23] Houser D, Keane M, McCabe K (2004). Behavior in a dynamic decision problem: An analysis of experimental evidence using a bayesian type classification algorithm.. Econometrica.

[24] Hampton AN, Bossaerts P, O'Doherty JP (2008). Neural correlates of mentalizing-related computations during strategic interactions in humans.. Proc Natl Acad Sci U S A.

[25] Dempster AP, Laird NM, Rubin DB (1977). Maximum likelihood from incomplete data via the EM algorithm.. J R Stat Soc Series B (Methodological).

[26] Greenberg E (2008). Introduction to Bayesian Econometrics.

[27] Diebolt J, Robert CP (1994). Estimation of finite mixture distributions through bayesian sampling.. J R Stat Soc Series B (Methodological).

[28] Lord C, Rutter M, Couteur A (1994). Autism diagnostic interview-revised: A revised version of a diagnostic interview for caregivers of individuals with possible pervasive developmental disorders.. J Autism Dev Disord.

[29] Rotter JB (1967). A new scale for the measurement of interpersonal trust.. J Pers.

[30] Trull TJ, Durrett CA (2005). Categorical and dimensional models of personality disorder.. Annu Rev Clin Psychol.

[31] Hubert L, Arabie P (1985). Comparing Partitions.. J Classif.

[32] Gentleman R, Ihaka R, Bates D, Chambers J, Dalgaard P (2007). R: A Language and Environment for Statistical Computing.. http://www.R-project.org.

[33] Fraley C, Raftery A (2008). Function adjustedRandIndex.. http://www.stat.washington.edu/fraley/mclust.

[34] American Psychiatric Association (2000). Diagnostic and statistical manual of mental disorders.

[35] Raftery AL, Lewis S, Bernardo JM, Berger JO, Dawid AP, Smith AFM (1992). How many iterations in the Gibbs sampler?. Bayesian Statistics 4.

[36] Smith BJ (2005). BOA: Bayesian Output Analysis Program, version 1.1.5.. http://www.public-health.uiowa.edu/boa/.

[37] Lewis SM, Raftery AE (1997). Estimating bayes factors via posterior simulation with the Laplace-Metropolis estimator.. J Am Stat Assoc.

[38] Gelman A, Carlin JB, Stern HS, Rubin DB (2003). Bayesian Data Analysis, Second Edition.

[39] Wilcoxon F (1945). Individual comparisons by ranking methods.. Biometrics Bulletin.

