# Supplementary material for: Biosensor Approach to Psychopathology Classification
Source: PLoS Comput Biol. 2010 Oct 21;6(10):e1000966. doi: 10.1371/journal.pcbi.1000966 (PMC2958801; doi:10.1371/journal.pcbi.1000966)
Supplement: Figure S1 — Mean ratios of investment and return for all dyads considered in analysis. Ratios of investment ( = MU/20) and return ( = MU/[3*investment amount]) are shown for each of the initial groups considered in the analysis. The number of pairs in each group is indicated in the title. Standard errors of the mean are displayed. (0.11 MB PDF) [file pcbi.1000966.s001.pdf]

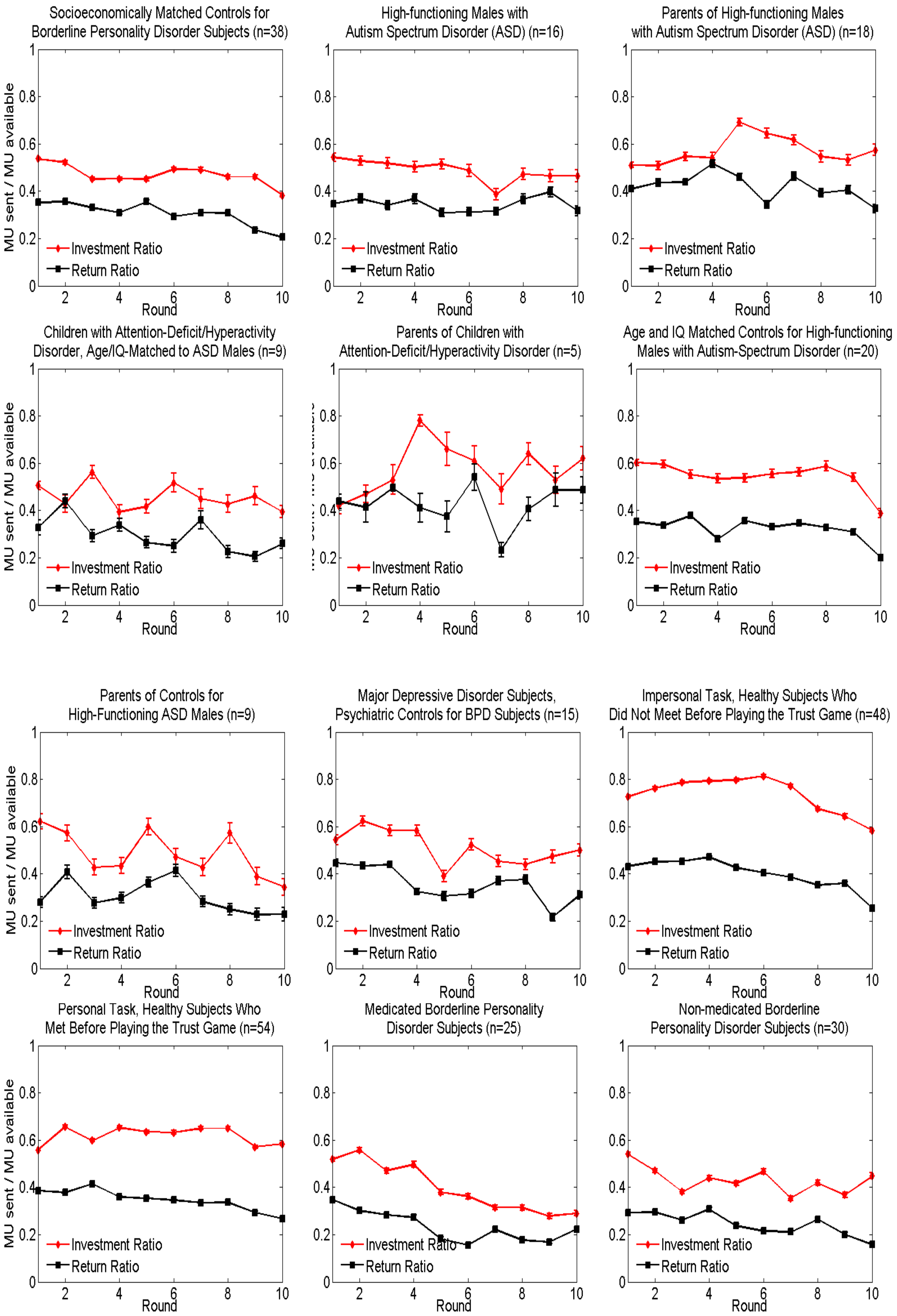
Socioeconomically Matched Controls for
Borderline Personality Disorder Subjects (n=38)
High-functioning Males with
Autism Spectrum Disorder (ASD) (n=16)
Parents of High-functioning Males
with Autism Spectrum Disorder (ASD) (n=18)
Children with Attention-Deficit/Hyperactivity
Disorder, Age/IQ-Matched to ASD Males (n=9)
Parents of Children with
Attention-Deficit/Hyperactivity Disorder (n=5)
Age and IQ Matched Controls for High-functioning
Males with Autism-Spectrum Disorder (n=20)
Parents of Controls for
High-Functioning ASD Males (n=9)
Major Depressive Disorder Subjects,
Psychiatric Controls for BPD Subjects (n=15)
Impersonal Task, Healthy Subjects Who
Did Not Meet Before Playing the Trust Game (n=48)
Personal Task, Healthy Subjects Who
Met Before Playing the Trust Game (n=54)
Medicated Borderline Personality
Disorder Subjects (n=25)
Non-medicated Borderline
Personality Disorder Subjects (n=30)
MU sent / MU available
Round
Investment Ratio
Return Ratio
