# Supplementary material for: Biosensor Approach to Psychopathology Classification
Source: PLoS Comput Biol. 2010 Oct 21;6(10):e1000966. doi: 10.1371/journal.pcbi.1000966 (PMC2958801; doi:10.1371/journal.pcbi.1000966)
Supplement: Figure S2 — Polynomial coefficient distributions over 30,000 draws from posterior distribution. The polynomial coefficients that predict investment ratios are stable after 30,000 draws from the posterior distribution and are approximately normally distributed. We show a histogram of each polynomial coefficient whose mean is shown in Fig 5. A red line is placed at the zero position, denoting a monomial that does not contribute to the value of the predicted investment ratio. (0.15 MB PDF) [file pcbi.1000966.s002.pdf]

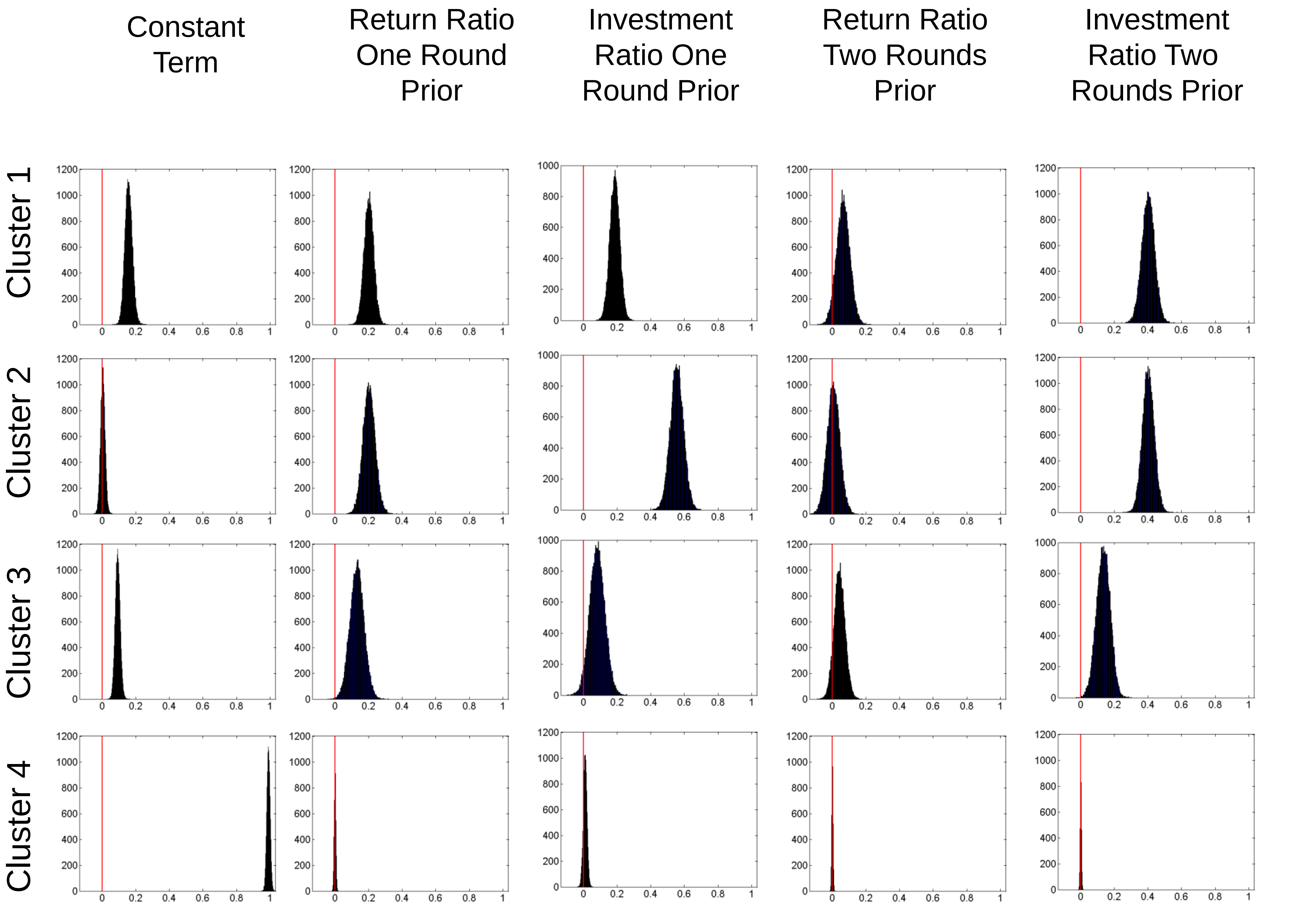
Constant Term
Return Ratio One Round Prior
Investment Ratio One Round Prior
Return Ratio Two Rounds Prior
Investment Ratio Two Rounds Prior
Cluster 1
Cluster 2
Cluster 3
Cluster 4
