# Supplementary figures and images for: Biosensor Approach to Psychopathology Classification
Source: PLoS Comput Biol. 2010 Oct 21;6(10):e1000966. doi: 10.1371/journal.pcbi.1000966 (PMC2958801; doi:10.1371/journal.pcbi.1000966)

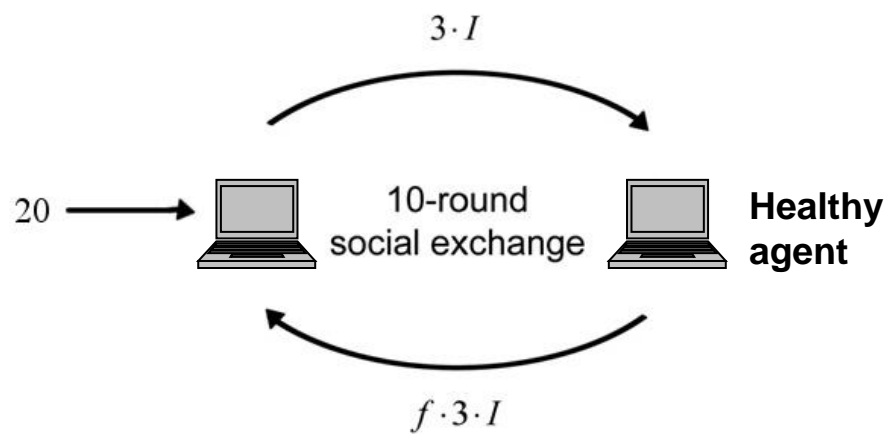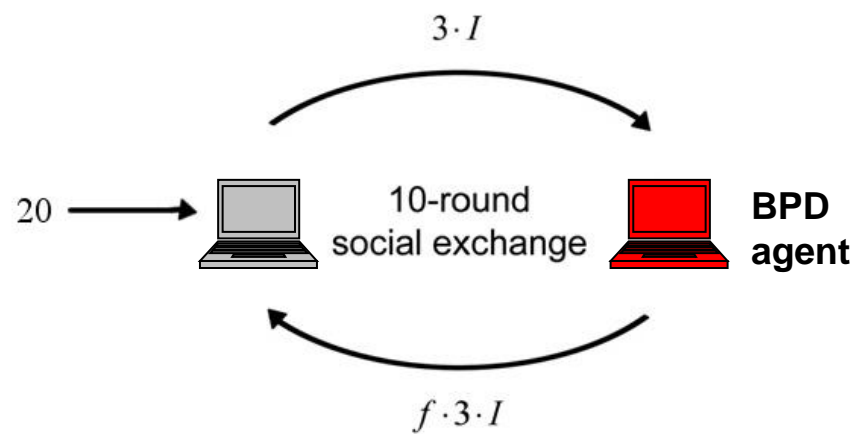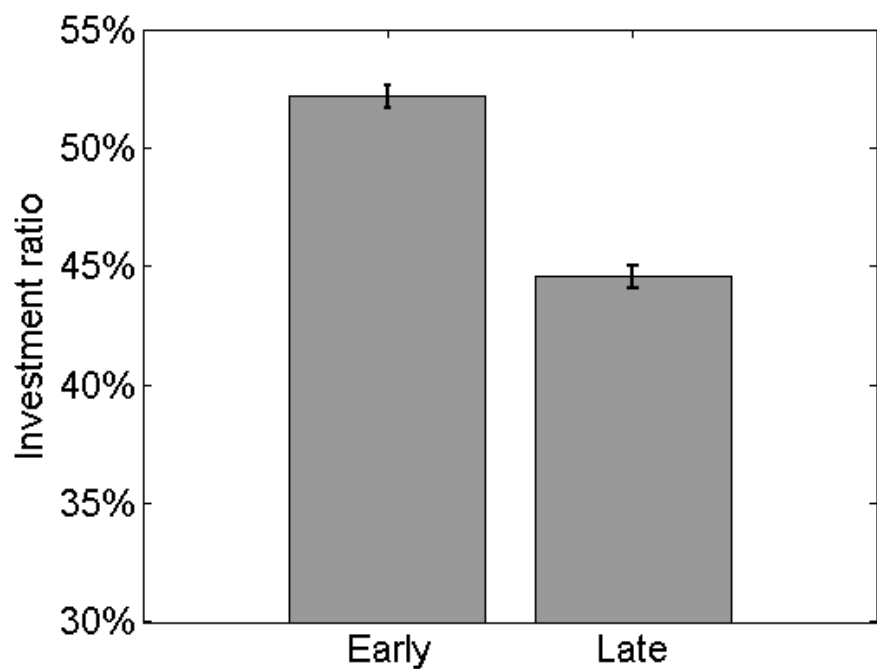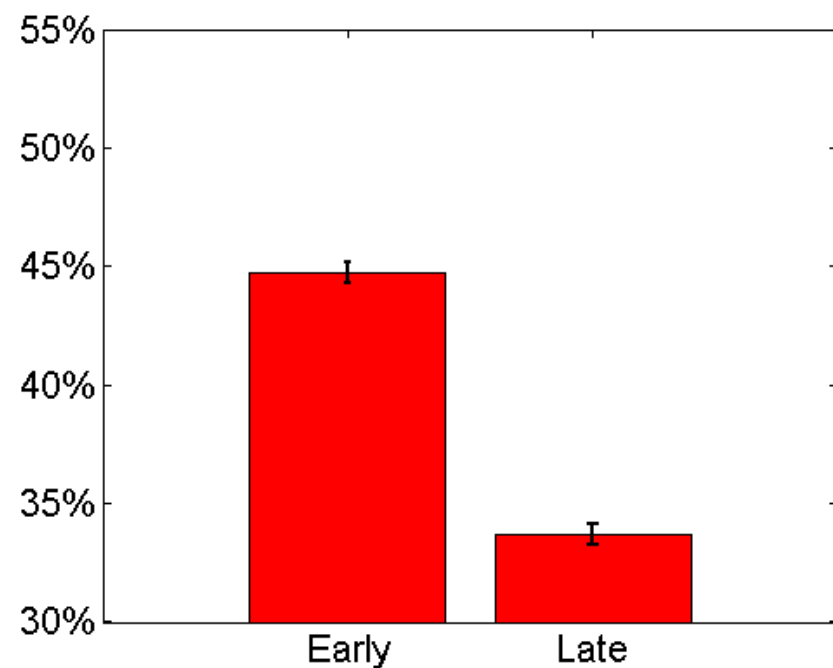

Supplement: Figure S3 — Mean ratios of investment for agent vs agent interactions. As seen in human players [6], cooperation fails across rounds when the BPD k-nearest neighbor sampling agent engages in a repeated exchange of trust with the control individual k-nearest neighbor sampling agent. Among 1,000 interactions between control agents (gray) paired with control trustees (gray), investments were large and sustained across early (1 to 5) rounds and late (6 to 10) rounds of the game. However, among 1,000 interactions between control agents (gray) paired with trustee agents sampling from interactions with BPD individuals (red), a decrease in investment level from early to late rounds of the game indicates a failure in cooperation across the iterated exchange. Mean percent invested and SEM are plotted. (0.03 MB PDF) [file pcbi.1000966.s003.pdf]

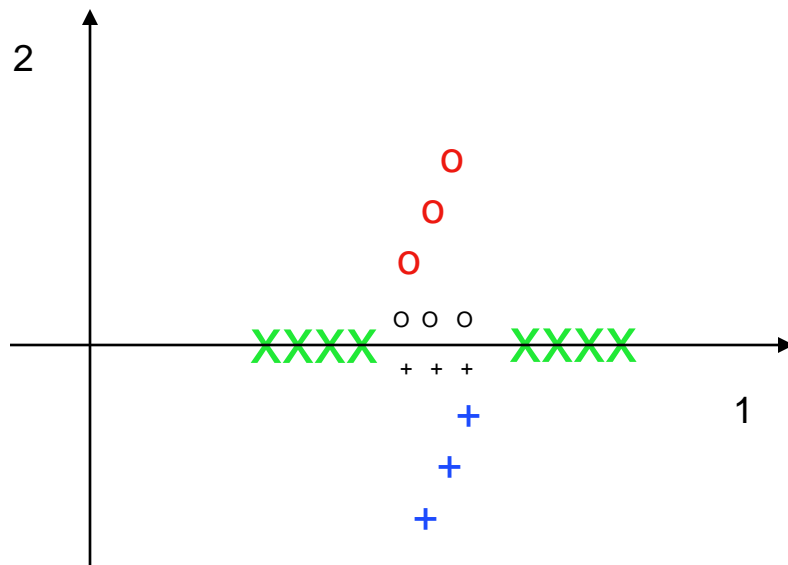

Supplement: Figure S4 — Clustering healthy versus healthy plus disordered dyads. Here X's represent healthy dyads, while the read and blue o's and +'s represent disordered dyads (the black and white + and −'s represent the projection onto the healthy dimensions). If the healthy dyads are clustered alone, then there would be two distinct clusters along the first axis. The disordered dyads would be assigned evenly across these clusters, resulting in no overrepresentation. In contrast, if all of the dyads are clustered, there would likely be two clusters, one with the o's overrepresented, and one with the +'s overrepresented. (0.00 MB PDF) [file pcbi.1000966.s004.pdf]

**Cluster 1 Betas**

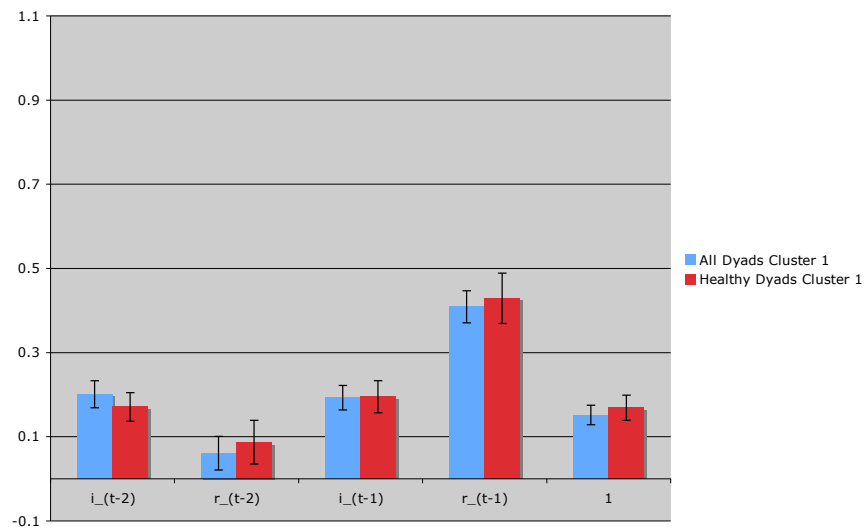

**Cluster 2 Betas**

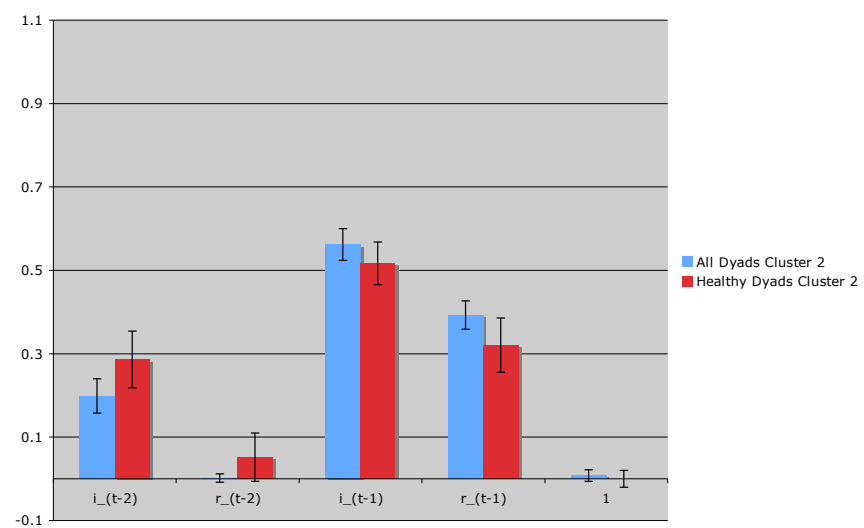

**Cluster 3 Betas**

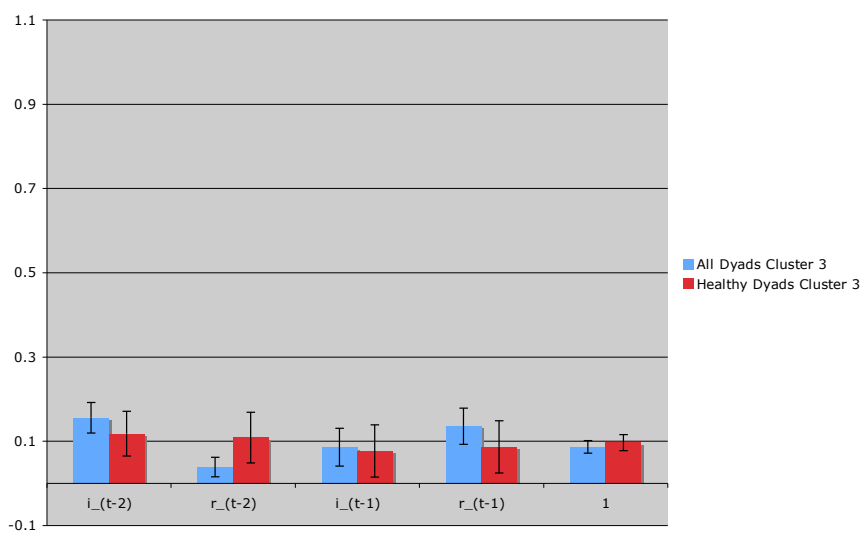

**Cluster 4 Betas**

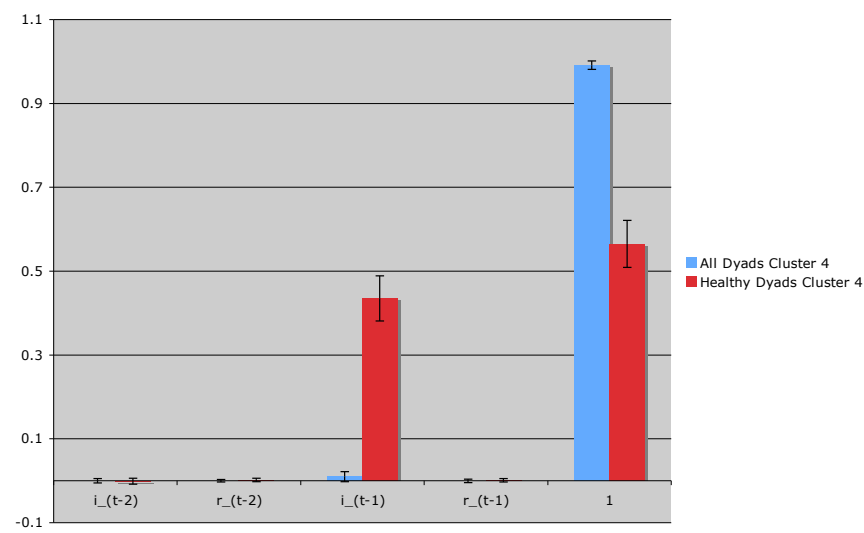

Supplement: Figure S5 — Means and standard deviations of posterior distributions of parameters in four clusters defined by clustering all dyads and healthy dyads only. (0.04 MB PDF) [file pcbi.1000966.s005.pdf]

A

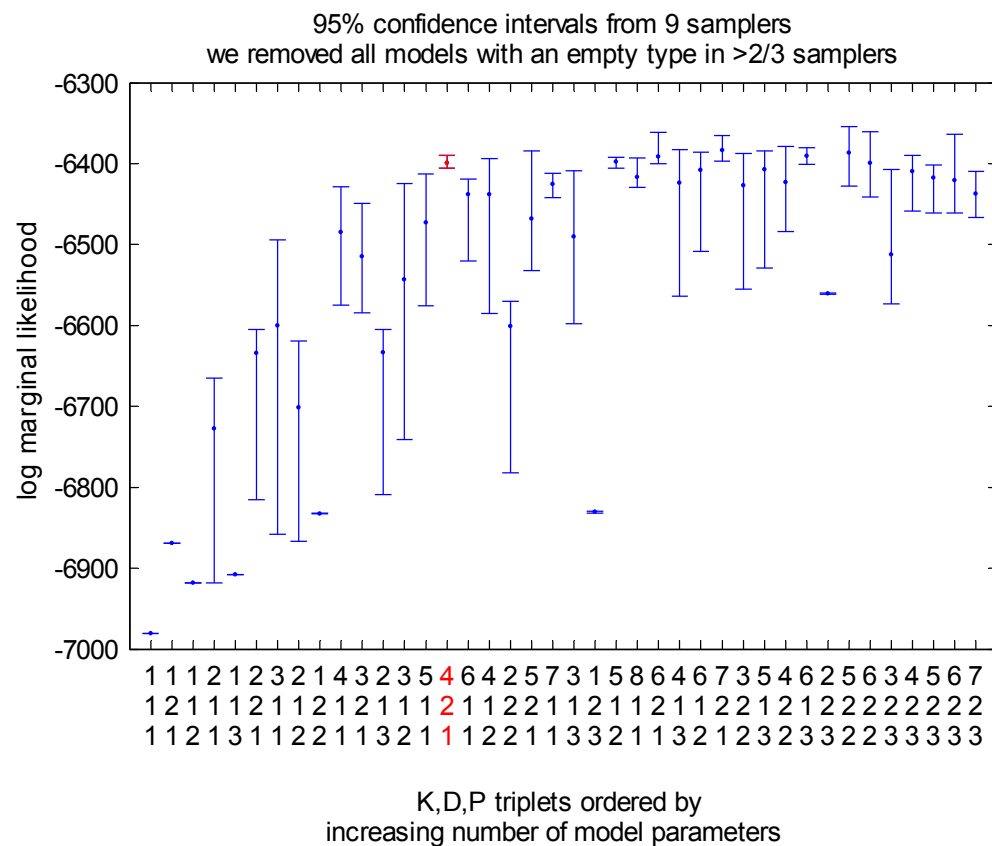

B

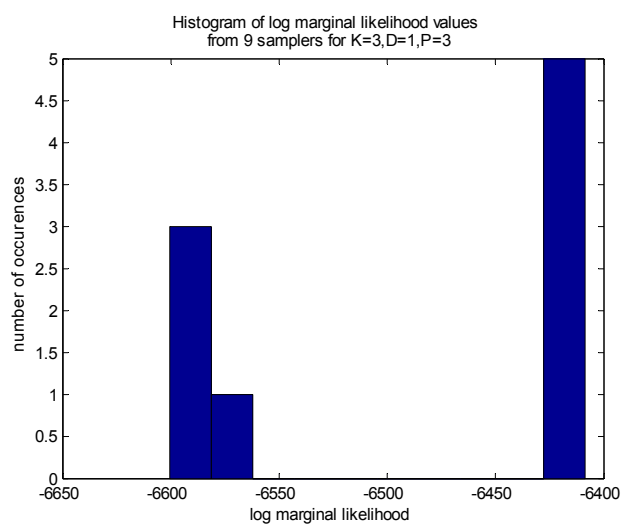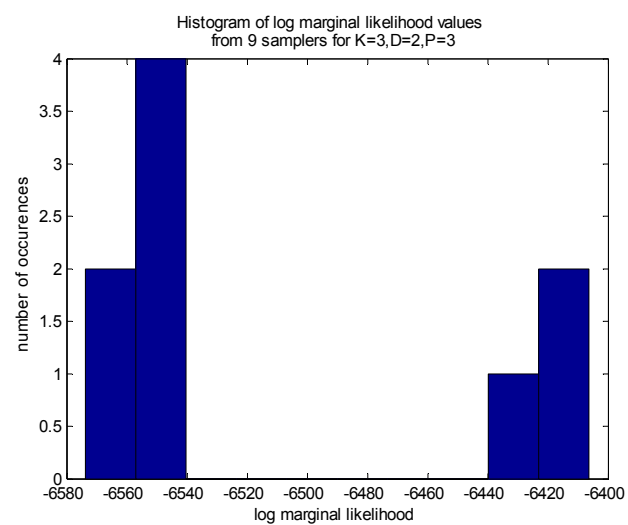

Supplement: Figure S6 — Model selection. A) Plot of the log marginal likelihoods computed using the method of Lewis-Raftery [37] used by Houser-Keane-McCabe [23]. We ran 9 samplers for each choice of the number of clusters K, number of rounds to look back D, and order of the polynomial P describing investment ratios in a given round in terms of ratios of investment and return in prior rounds. We used the standard quantile function in MATLAB R14 SP3 (Natick, MA) to compute 95% quantiles for our marginal likelihoods, which are plotted in this graph. B) Histograms of log marginal likelihood values for two samplers, showing that the distributions of the log marginal likelihood values are not Gaussian; thus, the Wilcoxon rank-sum test for comparison of medians was used [39]. (0.04 MB PDF) [file pcbi.1000966.s006.pdf]
