# Supplementary material for: Biosensor Approach to Psychopathology Classification
Source: PLoS Comput Biol. 2010 Oct 21;6(10):e1000966. doi: 10.1371/journal.pcbi.1000966 (PMC2958801; doi:10.1371/journal.pcbi.1000966)
Supplement: Table S1 — Available data. All trust game datasets used in the analysis are included here, along with appropriate references if applicable. The number of dyads in each dataset is shown, along with the abbreviations used in other figures and tables. (0.03 MB DOC) [file pcbi.1000966.s007.doc]

| Dyads | Abbreviation | Full Name |
| --- | --- | --- |
| 16 | AK | High-functioning Males with Autism Spectrum Disorder (ASD) [20] |
| 18 | AP | Parents of High-functioning Males with Autism Spectrum Disorder (ASD) [20] |
| 9 | HK | Children with Attention-Deficit/Hyperactivity Disorder; Psychiatric Controls for High-Functioning Males with Autism Spectrum Disorder |
| 5 | HP | Parents of Children with Attention-Deficit/Hyperactivity Disorder |
| 20 | CK | Age and IQ Matched Controls for High-functioning Males with Autism-Spectrum Disorder [20] |
| 9 | CP | Parents of Age and IQ Matched Controls for High-Functioning Males with Autism-Spectrum Disorder |
| 15 | MDD | Major Depressive Disorder Subjects; Psychiatric Controls for Borderline Personality Disorder Subjects |
| 48 | Imp | Impersonal Task; Healthy Subjects Who Did Not Meet Before Playing the Trust Game |
| 54 | Per | Personal Task; Healthy Subjects Who Met Before Playing the Trust Game |
| 25 | BPD-M | Medicated Borderline Personality Disorder Subjects [6] |
| 30 | BPD-N | Non-medicated Borderline Personality Disorder Subjects [6] |
| 38 | BPD-Ctrl | Socioeconomically Matched Controls for Borderline Personality Disorder Subjects [6] |
|  |  |  |
| 287 | All Dyads |  |
