# Supplementary material for: Biosensor Approach to Psychopathology Classification
Source: PLoS Comput Biol. 2010 Oct 21;6(10):e1000966. doi: 10.1371/journal.pcbi.1000966 (PMC2958801; doi:10.1371/journal.pcbi.1000966)
Supplement: Table S2 — Matching dyads to clusters. We present the most common and second most common match of all dyads in each original group into each new cluster. As can be seen, assignments are relatively stable over 30,000 draws from the posterior distribution (Fig. S2 shows the polynomial coefficient distributions for the same number of draws from the posterior distribution). (0.25 MB DOC) [file pcbi.1000966.s008.doc]

| Dyad Number (n) | Group | Most Common (Second Most Common) Cluster | Percent Match into Most Common (Second Most Common) Cluster | Dyad Number (n) | Group | Most Common (Second Most Common) Cluster | Percent Match into Most Common (Second Most Common) Cluster |
| --- | --- | --- | --- | --- | --- | --- | --- |
| 1 | ASD | 2 (3) | 80.16 (11.46)% | 41 | ADHD | 1 | 100.00% |
| 2 | ASD | 1 | 100.00% | 42 | ADHD | 1 (3) | 100.00 (0.00)% |
| 3 | ASD | 3 (2) | 92.07 (6.16)% | 43 | ADHD | 1 (2) | 97.87 (2.13)% |
| 4 | ASD | 2 (1) | 98.19 (1.81)% | 44 | HP | 1 (2) | 81.86 (18.14)% |
| 5 | ASD | 1 (3) | 78.98 (21.02)% | 45 | HP | 1 | 100.00% |
| 6 | ASD | 1 (2) | 99.84 (0.16)% | 46 | HP | 1 (2) | 100.00 (0.00)% |
| 7 | ASD | 2 (3) | 81.90 (17.85)% | 47 | HP | 1 | 100.00% |
| 8 | ASD | 1 | 100.00% | 48 | HP | 2 (1) | 53.99 (46.01)% |
| 9 | ASD | 1 (2) | 95.89 (3.38)% | 49 | CK | 1 (2) | 62.38 (37.62)% |
| 10 | ASD | 2 (1) | 94.57 (5.43)% | 50 | CK | 1 | 100.00% |
| 11 | ASD | 1 (2) | 91.29 (8.71)% | 51 | CK | 1 (2) | 99.28 (0.72)% |
| 12 | ASD | 2 (1) | 98.01 (1.99)% | 52 | CK | 4 | 100.00% |
| 13 | ASD | 2 (1) | 99.71 (0.29)% | 53 | CK | 4 | 100.00% |
| 14 | ASD | 1 (2) | 99.97 (0.03)% | 54 | CK | 3 (2) | 68.29 (21.77)% |
| 15 | ASD | 1 (2) | 57.68 (41.81)% | 55 | CK | 2 (1) | 98.28 (1.72)% |
| 16 | ASD | 2 (1) | 91.02 (8.98)% | 56 | CK | 1 | 100.00% |
| 17 | AP | 2 (1) | 99.31 (0.69)% | 57 | CK | 4 | 100.00% |
| 18 | AP | 1 | 100.00% | 58 | CK | 3 (2) | 61.60 (36.27)% |
| 19 | AP | 3 (2) | 68.73 (20.50)% | 59 | CK | 3 (2) | 93.05 (6.18)% |
| 20 | AP | 2 (1) | 99.91 (0.09)% | 60 | CK | 1 (2) | 96.35 (3.65)% |
| 21 | AP | 1 | 100.00% | 61 | CK | 3 (2) | 68.42 (23.30)% |
| 22 | AP | 3 (2) | 97.80 (1.68)% | 62 | CK | 1 (2) | 100.00 (0.00)% |
| 23 | AP | 1 (3) | 83.71 (16.27)% | 63 | CK | 1 (3) | 99.91 (0.07)% |
| 24 | AP | 1 (3) | 99.91 (0.05)% | 64 | CK | 1 | 100.00% |
| 25 | AP | 1 | 100.00% | 65 | CK | 2 (3) | 95.65 (3.09)% |
| 26 | AP | 1 (2) | 99.94 (0.06)% | 66 | CK | 1 | 100.00% |
| 27 | AP | 2 (1) | 99.87 (0.13)% | 67 | CK | 3 (1) | 93.84 (5.98)% |
| 28 | AP | 2 (1) | 88.48 (11.52)% | 68 | CK | 1 | 100.00% |
| 29 | AP | 2 (3) | 86.42 (13.11)% | 69 | CP | 2 (1) | 97.36 (1.54)% |
| 30 | AP | 4 | 100.00% | 70 | CP | 1 | 100.00% |
| 31 | AP | 3 (1) | 51.03 (48.87)% | 71 | CP | 1 (3) | 61.30 (37.63)% |
| 32 | AP | 1 (2) | 99.06 (0.94)% | 72 | CP | 1 | 100.00% |
| 33 | AP | 1 | 100.00% | 73 | CP | 2 (1) | 81.68 (18.32)% |
| 34 | AP | 1 (2) | 99.75 (0.25)% | 74 | CP | 1 | 100.00% |
| 35 | ADHD | 1 | 100.00% | 75 | CP | 2 (1) | 79.20 (20.80)% |
| 36 | ADHD | 1 | 100.00% | 76 | CP | 4 | 100.00% |
| 37 | ADHD | 1 (2) | 99.97 (0.03)% | 77 | CP | 1 (2) | 99.99 (0.01)% |
| 38 | ADHD | 1 (3) | 55.44 (42.64)% | 78 | MDD | 3 (1) | 87.33 (12.67)% |
| 39 | ADHD | 1 (2) | 99.99 (0.00)% | 79 | MDD | 1 (3) | 90.10 (8.98)% |
| 40 | ADHD | 2 (1) | 74.95 (18.49)% | 80 | MDD | 1 | 100.00% |

| Dyad Number (n) | Group | Most Common (Second Most Common) Cluster | Percent Match into Most Common (Second Most Common) Cluster | Dyad Number (n) | Group | Most Common (Second Most Common) Cluster | Percent Match into Most Common (Second Most Common) Cluster |
| --- | --- | --- | --- | --- | --- | --- | --- |
| 81 | MDD | 1 (2) | 99.81 (0.19)% | 121 | Imp | 4 | 100.00% |
| 82 | MDD | 3 (1) | 98.79 (1.18)% | 122 | Imp | 2 (1) | 99.82 (0.18)% |
| 83 | MDD | 1 | 100.00% | 123 | Imp | 4 | 100.00% |
| 84 | MDD | 4 | 100.00% | 124 | Imp | 1 | 100.00% |
| 85 | MDD | 1 | 100.00% | 125 | Imp | 2 (1) | 99.04 (0.96)% |
| 86 | MDD | 1 (3) | 99.72 (0.28)% | 126 | Imp | 3 (1) | 96.49 (3.10)% |
| 87 | MDD | 1 (2) | 80.77 (19.23)% | 127 | Imp | 2 (1) | 63.71 (36.29)% |
| 88 | MDD | 4 | 100.00% | 128 | Imp | 1 (2) | 100.00 (0.00)% |
| 89 | MDD | 1 (3) | 62.39 (33.34)% | 129 | Imp | 2 (1) | 98.84 (1.16)% |
| 90 | MDD | 2 (1) | 67.51 (32.48)% | 130 | Imp | 1 | 100.00% |
| 91 | MDD | 1 | 100.00% | 131 | Imp | 2 (1) | 84.74 (15.26)% |
| 92 | MDD | 4 | 100.00% | 132 | Imp | 1 (2) | 98.27 (1.73)% |
| 93 | Imp | 1 (2) | 99.92 (0.08)% | 133 | Imp | 2 (1) | 62.19 (37.81)% |
| 94 | Imp | 4 | 100.00% | 134 | Imp | 1 | 100.00% |
| 95 | Imp | 1 | 100.00% | 135 | Imp | 2 (1) | 99.45 (0.55)% |
| 96 | Imp | 1 | 100.00% | 136 | Imp | 2 (1) | 99.52 (0.48)% |
| 97 | Imp | 1 | 100.00% | 137 | Imp | 2 (1) | 99.64 (0.36)% |
| 98 | Imp | 1 | 100.00% | 138 | Imp | 2 (1) | 99.87 (0.13)% |
| 99 | Imp | 4 | 100.00% | 139 | Imp | 2 (1) | 99.45 (0.55)% |
| 100 | Imp | 1 | 100.00% | 140 | Imp | 2 (1) | 99.93 (0.07)% |
| 101 | Imp | 1 | 100.00% | 141 | Per | 1 | 100.00% |
| 102 | Imp | 1 | 100.00% | 142 | Per | 1 (2) | 76.50 (23.28)% |
| 103 | Imp | 1 | 100.00% | 143 | Per | 1 (2) | 99.56 (0.44)% |
| 104 | Imp | 1 (3) | 92.22 (7.46)% | 144 | Per | 1 | 100.00% |
| 105 | Imp | 4 | 100.00% | 145 | Per | 1 (3) | 86.72 (12.32)% |
| 106 | Imp | 1 | 100.00% | 146 | Per | 1 (2) | 89.03 (10.97)% |
| 107 | Imp | 1 | 100.00% | 147 | Per | 3 (1) | 95.88 (3.22)% |
| 108 | Imp | 3 (1) | 57.35 (42.39)% | 148 | Per | 4 | 100.00% |
| 109 | Imp | 1 | 100.00% | 149 | Per | 1 | 100.00% |
| 110 | Imp | 1 | 100.00% | 150 | Per | 2 (1) | 99.64 (0.36)% |
| 111 | Imp | 1 | 100.00% | 151 | Per | 4 | 100.00% |
| 112 | Imp | 3 (1) | 82.21 (17.79)% | 152 | Per | 2 (3) | 96.17 (2.66)% |
| 113 | Imp | 1 | 100.00% | 153 | Per | 1 | 100.00% |
| 114 | Imp | 1 | 100.00% | 154 | Per | 1 | 100.00% |
| 115 | Imp | 1 | 100.00% | 155 | Per | 1 | 100.00% |
| 116 | Imp | 2 (1) | 98.61 (1.39)% | 156 | Per | 1 (2) | 71.42 (28.58)% |
| 117 | Imp | 2 (1) | 98.56 (1.44)% | 157 | Per | 1 (2) | 99.99 (0.01)% |
| 118 | Imp | 1 (2) | 99.99 (0.01)% | 158 | Per | 4 | 100.00% |
| 119 | Imp | 2 (1) | 99.92 (0.08)% | 159 | Per | 4 | 100.00% |
| 120 | Imp | 2 (1) | 99.63 (0.37)% | 160 | Per | 1 (2) | 99.89 (0.11)% |

| Dyad Number (n) | Group | Most Common (Second Most Common) Cluster | Percent Match into Most Common (Second Most Common) Cluster | Dyad Number (n) | Group | Most Common (Second Most Common) Cluster | Percent Match into Most Common (Second Most Common) Cluster |
| --- | --- | --- | --- | --- | --- | --- | --- |
| 161 | Per | 1 (2) | 99.54 (0.46)% | 201 | BPD-M | 1 (2) | 81.22 (18.78)% |
| 162 | Per | 1 | 100.00% | 202 | BPD-M | 2 (1) | 95.55 (3.74)% |
| 163 | Per | 2 (1) | 99.84 (0.16)% | 203 | BPD-M | 2 (3) | 67.54 (32.37)% |
| 164 | Per | 1 (3) | 98.80 (1.19)% | 204 | BPD-M | 1 | 100.00% |
| 165 | Per | 1 | 100.00% | 205 | BPD-M | 3 (1) | 98.95 (0.97)% |
| 166 | Per | 1 | 100.00% | 206 | BPD-M | 1 (2) | 99.98 (0.02)% |
| 167 | Per | 2 (1) | 95.00 (5.00)% | 207 | BPD-M | 3 (2) | 97.29 (2.35)% |
| 168 | Per | 1 | 100.00% | 208 | BPD-M | 2 (3) | 95.60 (2.37)% |
| 169 | Per | 3 (2) | 74.96 (23.50)% | 209 | BPD-M | 2 (1) | 94.79 (5.21)% |
| 170 | Per | 4 | 100.00% | 210 | BPD-M | 3 (2) | 53.28 (44.07)% |
| 171 | Per | 1 (2) | 70.47 (29.53)% | 211 | BPD-M | 1 | 100.00% |
| 172 | Per | 1 (3) | 98.12 (1.88)% | 212 | BPD-M | 4 | 100.00% |
| 173 | Per | 1 | 100.00% | 213 | BPD-M | 2 (3) | 67.82 (31.30)% |
| 174 | Per | 1 (2) | 99.99 (0.01)% | 214 | BPD-M | 3 (2) | 99.40 (0.42)% |
| 175 | Per | 1 (2) | 99.87 (0.13)% | 215 | BPD-M | 1 (3) | 99.64 (0.36)% |
| 176 | Per | 4 | 100.00% | 216 | BPD-M | 3 (2) | 96.36 (3.52)% |
| 177 | Per | 4 | 100.00% | 217 | BPD-M | 1 (2) | 99.94 (0.06)% |
| 178 | Per | 2 (1) | 88.50 (7.65)% | 218 | BPD-M | 1 (2) | 99.49 (0.51)% |
| 179 | Per | 2 (1) | 98.79 (1.21)% | 219 | BPD-M | 3 (2) | 74.33 (20.30)% |
| 180 | Per | 2 (1) | 99.87 (0.13)% | 220 | BPD-N | 1 (2) | 97.94 (1.53)% |
| 181 | Per | 4 | 100.00% | 221 | BPD-N | 2 (1) | 84.59 (12.24)% |
| 182 | Per | 1 (2) | 99.72 (0.28)% | 222 | BPD-N | 1 (2) | 91.13 (8.69)% |
| 183 | Per | 1 | 100.00% | 223 | BPD-N | 3 (2) | 56.52 (42.13)% |
| 184 | Per | 3 (2) | 66.86 (33.06)% | 224 | BPD-N | 2 (3) | 65.49 (33.53)% |
| 185 | Per | 2 (1) | 98.28 (1.11)% | 225 | BPD-N | 2 (1) | 99.75 (0.25)% |
| 186 | Per | 1 (2) | 99.93 (0.07)% | 226 | BPD-N | 2 (3) | 38.30 (32.08)% |
| 187 | Per | 1 | 100.00% | 227 | BPD-N | 3 (1) | 85.12 (11.77)% |
| 188 | Per | 1 (2) | 99.70 (0.30)% | 228 | BPD-N | 1 (3) | 60.04 (39.84)% |
| 189 | Per | 1 (2) | 96.36 (3.64)% | 229 | BPD-N | 1 | 100.00% |
| 190 | Per | 1 | 100.00% | 230 | BPD-N | 1 (3) | 69.78 (18.78)% |
| 191 | Per | 3 (2) | 66.70 (32.67)% | 231 | BPD-N | 3 (2) | 98.18 (1.55)% |
| 192 | Per | 2 (1) | 89.45 (10.55)% | 232 | BPD-N | 2 (1) | 89.82 (10.18)% |
| 193 | Per | 4 | 100.00% | 233 | BPD-N | 3 (1) | 55.46 (37.30)% |
| 194 | Per | 3 (1) | 99.05 (0.86)% | 234 | BPD-N | 3 (2) | 63.34 (24.30)% |
| 195 | BPD-M | 1 (2) | 82.02 (17.50)% | 235 | BPD-N | 3 (1) | 86.81 (7.91)% |
| 196 | BPD-M | 3 (1) | 62.21 (29.85)% | 236 | BPD-N | 1 (2) | 100.00 (0.00)% |
| 197 | BPD-M | 2 (3) | 93.07 (5.55)% | 237 | BPD-N | 3 (2) | 77.45 (21.41)% |
| 198 | BPD-M | 1 | 100.00% | 238 | BPD-N | 3 (1) | 95.50 (4.29)% |
| 199 | BPD-M | 1 | 100.00% | 239 | BPD-N | 1 | 100.00% |
| 200 | BPD-M | 3 (2) | 75.22 (24.43)% | 240 | BPD-N | 1 (2) | 99.99 (0.01)% |

| Dyad Number (n) | Group | Most Common (Second Most Common) Cluster | Percent Match into Most Common (Second Most Common) Cluster | Dyad Number (n) | Group | Most Common (Second Most Common) Cluster | Percent Match into Most Common (Second Most Common) Cluster |
| --- | --- | --- | --- | --- | --- | --- | --- |
| 241 | BPD-N | 1 (2) | 100.00 (0.00)% | 281 | BPD-Ctrl | 1 (3) | 82.27 (16.96)% |
| 242 | BPD-N | 2 (1) | 99.88 (0.12)% | 282 | BPD-Ctrl | 3 (2) | 99.33 (0.65)% |
| 243 | BPD-N | 1 | 100.00% | 283 | BPD-Ctrl | 1 (2) | 99.68 (0.32)% |
| 244 | BPD-N | 1 (3) | 98.42 (1.32)% | 284 | BPD-Ctrl | 1 (2) | 99.86 (0.14)% |
| 245 | BPD-N | 2 (1) | 91.98 (8.02)% | 285 | BPD-Ctrl | 3 (2) | 97.17 (2.07)% |
| 246 | BPD-N | 2 (1) | 95.66 (4.07)% | 286 | BPD-Ctrl | 3 (2) | 75.94 (18.34)% |
| 247 | BPD-N | 2 (1) | 91.00 (8.98)% | 287 | BPD-Ctrl | 2 (1) | 99.57 (0.43)% |
| 248 | BPD-N | 1 | 100.00% |  |  |  |  |
| 249 | BPD-N | 2 (1) | 99.78 (0.22)% |  |  |  |  |
| 250 | BPD-Ctrl | 1 | 100.00% |  |  |  |  |
| 251 | BPD-Ctrl | 1 (2) | 100.00 (0.00)% |  |  |  |  |
| 252 | BPD-Ctrl | 1 | 100.00% |  |  |  |  |
| 253 | BPD-Ctrl | 2 (3) | 94.94 (4.05)% |  |  |  |  |
| 254 | BPD-Ctrl | 1 (2) | 99.96 (0.04)% |  |  |  |  |
| 255 | BPD-Ctrl | 1 (3) | 72.00 (27.65)% |  |  |  |  |
| 256 | BPD-Ctrl | 2 (1) | 96.99 (2.99)% |  |  |  |  |
| 257 | BPD-Ctrl | 1 (2) | 98.27 (1.69)% |  |  |  |  |
| 258 | BPD-Ctrl | 1 (3) | 99.93 (0.07)% |  |  |  |  |
| 259 | BPD-Ctrl | 1 (2) | 85.49 (14.51)% |  |  |  |  |
| 260 | BPD-Ctrl | 1 (2) | 94.36 (5.64)% |  |  |  |  |
| 261 | BPD-Ctrl | 1 | 100.00% |  |  |  |  |
| 262 | BPD-Ctrl | 1 | 100.00% |  |  |  |  |
| 263 | BPD-Ctrl | 1 (2) | 50.33 (49.67)% |  |  |  |  |
| 264 | BPD-Ctrl | 3 (2) | 93.45 (6.43)% |  |  |  |  |
| 265 | BPD-Ctrl | 2 (1) | 94.62 (5.38)% |  |  |  |  |
| 266 | BPD-Ctrl | 2 (1) | 99.33 (0.67)% |  |  |  |  |
| 267 | BPD-Ctrl | 3 (2) | 54.64 (45.13)% |  |  |  |  |
| 268 | BPD-Ctrl | 1 | 100.00% |  |  |  |  |
| 269 | BPD-Ctrl | 1 | 100.00% |  |  |  |  |
| 270 | BPD-Ctrl | 1 (2) | 99.99 (0.01)% |  |  |  |  |
| 271 | BPD-Ctrl | 1 | 100.00% |  |  |  |  |
| 272 | BPD-Ctrl | 2 (1) | 99.37 (0.63)% |  |  |  |  |
| 273 | BPD-Ctrl | 1 (3) | 99.44 (0.43)% |  |  |  |  |
| 274 | BPD-Ctrl | 1 (3) | 87.03 (12.94)% |  |  |  |  |
| 275 | BPD-Ctrl | 4 | 100.00% |  |  |  |  |
| 276 | BPD-Ctrl | 3 (1) | 92.94 (7.06)% |  |  |  |  |
| 277 | BPD-Ctrl | 1 | 100.00% |  |  |  |  |
| 278 | BPD-Ctrl | 3 (1) | 68.63 (17.78)% |  |  |  |  |
| 279 | BPD-Ctrl | 1 | 100.00% |  |  |  |  |
| 280 | BPD-Ctrl | 1 | 100.00% |  |  |  |  |
