# Supplementary material for: Biosensor Approach to Psychopathology Classification
Source: PLoS Comput Biol. 2010 Oct 21;6(10):e1000966. doi: 10.1371/journal.pcbi.1000966 (PMC2958801; doi:10.1371/journal.pcbi.1000966)
Supplement: Table S3 — Groups over-/under-represented in behavioral clusters. We present the number and percentage of each original group matched into each new cluster. The assignment, as in previous tables, is based on the most common match of each dyad over 30,000 draws from the posterior distribution. (0.04 MB DOC) [file pcbi.1000966.s009.doc]

| Abbreviation |  | Dyads  in Cluster 1 | Dyads  in Cluster 2 | Dyads  in Cluster 3 | Dyads  in Cluster 4 |
| --- | --- | --- | --- | --- | --- |
| ASD |  | 50% | 44% | 06% | 00% |
| AP |  | 50% | 28% | 17% | 06% |
| ADHD |  | 89% | 11% | 00% | 00% |
| HP |  | 80% | 20% | 00% | 00% |
| CK |  | 50% | 10% | 25% | 15% |
| CP |  | 56% | 33% | 00% | 11% |
| MDD |  | 60% | 07% | 13% | 20% |
| Imp |  | 50% | 33% | 06% | 10% |
| Per |  | 57% | 17% | 09% | 07% |
| BPD-M |  | 40% | 24% | 32% | 00% |
| BPD-N |  | 40% | 33% | 27% | 00% |
| BPD-Controls |  | 63% | 16% | 18% | 03% |
|  |  |  |  |  |  |
| All Dyads |  | 54% | 23% | 15% | 08% |
