# Supplementary material for: Biosensor Approach to Psychopathology Classification
Source: PLoS Comput Biol. 2010 Oct 21;6(10):e1000966. doi: 10.1371/journal.pcbi.1000966 (PMC2958801; doi:10.1371/journal.pcbi.1000966)
Supplement: Table S4 — Model selection. We present log marginal likelihoods estimated using the method of Lewis-Raftery [37] from 9 samplers for each choice of number of clusters K, look-back rounds D, and order of polynomial P describing the dependence of investment ratios in a given round on investment & return ratios in prior rounds. We sort all models by the number of model parameters, and discard models for which > = 6 have an empty type in the mode of all 5,000 draws from the posterior (after the first 3,000 is discarded as burn-in). We used the Wilcoxon Rank-Sum Test [39] to compare a given model's median log marginal likelihood with that of each model with fewer parameters. We chose the model (red) with the largest marginal likelihood for which we can guarantee that it is better than all parsimonious models. We report, in the right-hand column, for each model, the number of the first model for which we cannot guarantee the marginal likelihood is superior; that is, either (i) the median of this model is lower than the median of the model # in this column or (ii) the Wilcoxon rank-sum test, as implemented in MATLAB R14 SP3 (Natick, MA), does not reject the null hypothesis that the medians of the log marginal likelihoods for the two different models come from the same distribution at a 95% significance level. Please also see Figure S5. For the case of two models: K = 4, D = 2, P = 1 and K = 3, D = 2, P = 1, we performed an analysis using 3 samplers to compare the method of marginal likelihood used above based on the posterior mode [25] with a method, the mean harmonic estimator, that is based on using all draws from the posterior (see [38] for a detailed review of this and other methods of calculating marginal likelihoods and model selection). We found, for the median value of 3 samplers for each model, the log marginal likelihood for the Lewis-Raftery method preferred the K = 4, D = 2, P = 1 model by 36.64 units, whereas the mean harmonic estimator preferred the K = 4, D = 2, P = 1 [file pcbi.1000966.s010.doc]

| model | log marginal likelihoods from 9 samplers for each model | median | **K** | **D** | **P** | parameters | see legend |
| --- | --- | --- | --- | --- | --- | --- | --- |
| 2 | -6869.35,-6869.33,-6869.35,-6869.32,-6869.26,-6869.24,-6869.35,-6869.29,-6869.32 | -6869.32 | **1** | **2** | **1** | 6 |  |
| 3 | -6918.1,-6917.96,-6917.94,-6918,-6917.93,-6917.88,-6917.92,-6917.98,-6917.99 | -6917.96 | **1** | **1** | **2** | 7 | 2 |
| 4 | -6674.39,-6663.06,-6674.41,-6674.41,-6918.65,-6674.46,-6674.27,-6674.23,-6917.83 | -6674.41 | **2** | **1** | **1** | 9 |  |
| 5 | -6907.66,-6907.73,-6907.68,-6907.45,-6907.61,-6907.68,-6907.69,-6907.64,-6907.77 | -6907.68 | **1** | **1** | **3** | 11 | 2 |
| 6 | -6605.47,-6605.64,-6604.81,-6605.52,-6605.11,-6605.05,-6605.42,-6605.26,-6867.88 | -6605.42 | **2** | **2** | **1** | 13 |  |
| 7 | -6495.89,-6556,-6557.51,-6913.85,-6635.52,-6636.38,-6554.62,-6493.48,-6556.46 | -6556.46 | **3** | **1** | **1** | 14 | 6 |
| 8 | -6619.71,-6619.92,-6867.38,-6619.42,-6620.05,-6619.82,-6619.79,-6865.08,-6865.03 | -6619.92 | **2** | **1** | **2** | 15 | 4 |
| 9 | -6832.36,-6832.68,-6832.46,-6832.42,-6832.63,-6832.86,-6832.76,-6832.3,-6832.84 | -6832.63 | **1** | **2** | **2** | 16 | 4 |
| 10 | -6427.36,-6530.05,-6433.03,-6583.7,-6432.03,-6531.77,-6537.96,-6442.53,-6440.28 | -6442.53 | **4** | **1** | **1** | 19 |  |
| 11 | -6505.9,-6506.47,-6436.34,-6506.19,-6508.04,-6504.23,-6583.65,-6584.28,-6500.39 | -6506.19 | **3** | **2** | **1** | 20 | 7 |
| 12 | -6605.12,-6604.98,-6604.5,-6605.9,-6605.18,-6605.7,-6605.63,-6860.19,-6605.3 | -6605.30 | **2** | **1** | **3** | 23 | 6 |
| 13 | -6424.2,-6614.14,-6772.61,-6587.4,-6427.22,-6596.02,-6424.65,-6427.03,-6614.31 | -6587.40 | **3** | **1** | **2** | 23 | 6 |
| 14 | -6418.91,-6532.93,-6574.76,-6438.75,-6432.15,-6575.86,-6439.48,-6430.91,-6411.37 | -6438.75 | **5** | **1** | **1** | 24 | 10 |
| **15** | ***-6394.97,-6402.28,-6397.25,-6398.98,-6388.2,-6398.96,-6405.82,-6403.8,-6403.27*** | ***-6398.98*** | ***4*** | ***2*** | ***1*** | ***27*** |  |
| 16 | -6418.46,-6429.55,-6426.22,-6425.28,-6424.05,-6425.1,-6422.71,-6542.72,-6425.73 | -6425.28 | **6** | **1** | **1** | 29 | 14 |
| 17 | -6393.41,-6402.5,-6398.13,-6394.09,-6397.21,-6586.18,-6394.93,-6580.07,-6398.16 | -6398.13 | **4** | **1** | **2** | 31 | 14 |
| 18 | -6572.52,-6834.41,-6571.42,-6572.13,-6572.58,-6572.13,-6571.53,-6570.31,-6571.01 | -6572.13 | **2** | **2** | **2** | 33 | 7 |
| 19 | -6531.11,-6382.06,-6394.83,-6532.54,-6531.1,-6490.88,-6502.7,-6445.61,-6398.95 | -6490.88 | **5** | **2** | **1** | 34 | 10 |
| 20 | -6412.78,-6411.36,-6427.59,-6421.64,-6430.98,-6418.6,-6442.86,-6419.65,-6439.67 | -6421.64 | **7** | **1** | **1** | 34 | 14 |
| 21 | -6577.89,-6588.08,-6408.6,-6409.47,-6600.43,-6588.03,-6416.8,-6411.15,-6411.63 | -6416.80 | **3** | **1** | **3** | 35 | 7 |
| 22 | -6829.74,-6830.36,-6829.3,-6830.03,-6831.03,-6831.69,-6831.38,-6829.88,-6831.25 | -6830.36 | **1** | **2** | **3** | 36 | 4 |
| 23 | -6398.34,-6397.98,-6391.37,-6397.78,-6398.13,-6398.24,-6395.2,-6407.47,-6395.66 | -6397.98 | **5** | **1** | **2** | 39 | 15 |
| 24 | -6389.94,-6426.58,-6430.36,-6417.81,-6422.39,-6413.22,-6404.66,-6426.1,-6418.12 | -6418.12 | **8** | **1** | **1** | 39 | 15 |
| 25 | -6400.26,-6398.02,-6396.41,-6398.42,-6394,-6398,-6388.54,-6354.36,-6393.57 | -6396.41 | **6** | **2** | **1** | 41 | 15 |
| 26 | -6386.06,-6385.48,-6387.33,-6390.01,-6569.57,-6385.49,-6381.48,-6386.35,-6541.18 | -6386.35 | **4** | **1** | **3** | 47 | 15 |
| 27 | -6535.23,-6386.35,-6400.57,-6392.09,-6390.75,-6391.67,-6385.33,-6391.51,-6396.62 | -6391.67 | **6** | **1** | **2** | 47 | 15 |
| 28 | -6381.1,-6393.53,-6389.82,-6370.86,-6397.77,-6390.63,-6363.37,-6388.3,-6373.23 | -6388.30 | **7** | **2** | **1** | 48 | 26 |
| 29 | -6387.02,-6557.77,-6392.02,-6545.59,-6388.86,-6390.97,-6390.46,-6393.58,-6395.14 | -6392.02 | **3** | **2** | **2** | 50 | 15 |
| 30 | -6392.68,-6385.96,-6388.81,-6383.66,-6562.86,-6386.08,-6387.98,-6388.53,-6389.39 | -6388.53 | **5** | **1** | **3** | 59 | 25 |
| 31 | -6380.42,-6483.82,-6449.14,-6480.9,-6386.38,-6378.26,-6387.41,-6482.65,-6379.66 | -6387.41 | **4** | **2** | **2** | 67 | 10 |
| 32 | -6398.61,-6385.49,-6397.57,-6388.91,-6379.53,-6401.64,-6384.43,-6395.33,-6381.33 | -6388.91 | **6** | **1** | **3** | 71 | 17 |
| 33 | -6560.16,-6560.63,-6560.47,-6560.07,-6561.13,-6560.13,-6559.33,-6561.56,-6560.44 | -6560.44 | **2** | **2** | **3** | 73 | 7 |
| 34 | -6379.34,-6436.04,-6377.28,-6347.96,-6395.76,-6382.74,-6392.94,-6386.83,-6378.7 | -6382.74 | **5** | **2** | **2** | 84 | 25 |
| 35 | -6436.85,-6399.89,-6373.42,-6380.91,-6441.38,-6357.38,-6380.82,-6383.21,-6438.61 | -6383.21 | **6** | **2** | **2** | 101 | 15 |
| 36 | -6408.99,-6552.37,-6406.4,-6437.78,-6551.93,-6553.87,-6573.84,-6554.7,-6572.83 | -6552.37 | **3** | **2** | **3** | 110 | 7 |
| 37 | -6398.01,-6398.13,-6396.53,-6389.39,-6457.6,-6458.35,-6400.91,-6397.37,-6391.89 | -6398.01 | **4** | **2** | **3** | 147 | 15 |
| 38 | -6445.03,-6406.65,-6404.92,-6465.43,-6413,-6413.96,-6401.83,-6405.82,-6401.76 | -6406.65 | **5** | **2** | **3** | 184 | 15 |
| 39 | -6426.84,-6422.81,-6460.86,-6423.5,-6355.72,-6394.85,-6420,-6461.27,-6419.74 | -6422.81 | **6** | **2** | **3** | 221 | 14 |
| 40 | -6431.16,-6430.51,-6426.65,-6465.8,-6430.62,-6438.15,-6467.03,-6441.04,-6405.73 | -6431.16 | **7** | **2** | **3** | 258 | 10 |
