# Supplementary material for: Biosensor Approach to Psychopathology Classification
Source: PLoS Comput Biol. 2010 Oct 21;6(10):e1000966. doi: 10.1371/journal.pcbi.1000966 (PMC2958801; doi:10.1371/journal.pcbi.1000966)
Supplement: Table S5 — Degree of clustering expressed in terms of clinically relevant indices. For each of the four pathologies, this table describes the values of the standard clinically relevant indices: sensitivity, specificity, and positive and negative predictive values. (0.02 MB DOC) [file pcbi.1000966.s011.doc]

Group Cluster Sensitivity Specificity Positive Negative Number Predictive Predictive Value Value

ADHD 1 0.89 0.47 0.05 0.99

ASD 2 0.44 0.78 0.11 0.96

BPD 3 0.31 0.89 0.40 0.84

MDD 4 0.27 0.93 0.17 0.96
