# Supplementary material for: Biosensor Approach to Psychopathology Classification
Source: PLoS Comput Biol. 2010 Oct 21;6(10):e1000966. doi: 10.1371/journal.pcbi.1000966 (PMC2958801; doi:10.1371/journal.pcbi.1000966)
Supplement: Table S6 — Clustering based on all the trustees vs. clustering based only on healthy trustees. For each of the four pathologies, this table describes the over-representation of participants with this pathology in the corresponding cluster. According to our computations (see Methods section), only over-estimations of 1.5 and larger are statistically significant. (0.02 MB DOC) [file pcbi.1000966.s012.doc]

Group Cluster Over-representation based on Over-representation based on Number all trustees healthy trustees only

ADHD 1 1.50 1.17

ASD 2 1.76 0.97

BPD-M 3 2.85 3.65

BPD-N 3 1.69 3.63

MDD 4 2.55 0.42
