# Supplementary material for: Biosensor Approach to Psychopathology Classification
Source: PLoS Comput Biol. 2010 Oct 21;6(10):e1000966. doi: 10.1371/journal.pcbi.1000966 (PMC2958801; doi:10.1371/journal.pcbi.1000966)
Supplement: Table S7 — Summary statistics of posterior distributions of regression coefficients. (0.46 MB DOC) [file pcbi.1000966.s013.doc]

Summary Statistics of Posterior Distributions of Regression Coefficients

Clusters Estimated Using All Dyads

Cluster 1

| Parameter | N | Mean | Standard  Deviation | 25% | 50% | 75% |
| --- | --- | --- | --- | --- | --- | --- |
| Constant | 5000 | 0.157 | 0.022 | 0.142 | 0.156 | 0.172 |
| $r_{t-1}$ | 5000 | 0.402 | 0.038 | 0.377 | 0.402 | 0.428 |
| $i_{t-1}$ | 5000 | 0.185 | 0.030 | 0.165 | 0.185 | 0.206 |
| $r_{t-2}$ | 5000 | 0.065 | 0.040 | 0.037 | 0.065 | 0.092 |
| $i_{t-2}$ | 5000 | 0.201 | 0.028 | 0.182 | 0.202 | 0.221 |
| $\sigma^{2}$ | 5000 | 0.288 | 0.006 | 0.283 | 0.288 | 0.292 |

Cluster 2

| Parameter | N | Mean | Standard  Deviation | 25% | 50% | 75% |
| --- | --- | --- | --- | --- | --- | --- |
| Constant | 5000 | 0.003 | 0.013 | -0.006 | 0.003 | 0.012 |
| $r_{t-1}$ | 5000 | 0.403 | 0.035 | 0.379 | 0.403 | 0.427 |
| $i_{t-1}$ | 5000 | 0.556 | 0.040 | 0.529 | 0.556 | 0.582 |
| $r_{t-2}$ | 5000 | 0.007 | 0.037 | -0.019 | 0.007 | 0.032 |
| $i_{t-2}$ | 5000 | 0.201 | 0.037 | 0.176 | 0.201 | 0.226 |
| $\sigma^{2}$ | 5000 | 0.119 | 0.004 | 0.116 | 0.119 | 0.121 |

Cluster 3

| Parameter | N | Mean | Standard  Deviation | 25% | 50% | 75% |
| --- | --- | --- | --- | --- | --- | --- |
| Constant | 5000 | 0.092 | 0.014 | 0.082 | 0.092 | 0.102 |
| $r_{t-1}$ | 5000 | 0.134 | 0.040 | 0.107 | 0.134 | 0.162 |
| $i_{t-1}$ | 5000 | 0.082 | 0.044 | 0.051 | 0.082 | 0.112 |
| $r_{t-2}$ | 5000 | 0.043 | 0.032 | 0.021 | 0.043 | 0.065 |
| $i_{t-2}$ | 5000 | 0.131 | 0.042 | 0.102 | 0.131 | 0.157 |
| $\sigma^{2}$ | 5000 | 0.117 | 0.005 | 0.114 | 0.117 | 0.120 |

Cluster 4

| Parameter | N | Mean | Standard  Deviation | 25% | 50% | 75% |
| --- | --- | --- | --- | --- | --- | --- |
| Constant | 5000 | 0.991 | 0.010 | 0.985 | 0.991 | 0.998 |
| $r_{t-1}$ | 5000 | 0.000 | 0.003 | -0.002 | 0.000 | 0.003 |
| $i_{t-1}$ | 5000 | 0.009 | 0.011 | 0.002 | 0.009 | 0.016 |
| $r_{t-2}$ | 5000 | 0.000 | 0.004 | -0.002 | 0.000 | 0.002 |
| $i_{t-2}$ | 5000 | 0.000 | 0.005 | -0.003 | 0.000 | 0.003 |
| $\sigma^{2}$ | 5000 | 0.007 | 0.000 | 0.007 | 0.007 | 0.008 |

Summary Statistics of Posterior Distributions of Regression Coefficients

Clusters Estimated Using Healthy Dyads Only

Cluster 1

| Parameter | N | Mean | Standard  Deviation | 25% | 50% | 75% |
| --- | --- | --- | --- | --- | --- | --- |
| Constant | 5000 | 0.172 | 0.028 | 0.153 | 0.171 | 0.190 |
| $r_{t-1}$ | 5000 | 0.428 | 0.049 | 0.396 | 0.429 | 0.460 |
| $i_{t-1}$ | 5000 | 0.200 | 0.037 | 0.175 | 0.201 | 0.226 |
| $r_{t-2}$ | 5000 | 0.085 | 0.052 | 0.050 | 0.086 | 0.119 |
| $i_{t-2}$ | 5000 | 0.167 | 0.037 | 0.142 | 0.168 | 0.192 |
| $\sigma^{2}$ | 5000 | 0.292 | 0.008 | 0.286 | 0.291 | 0.297 |

Cluster 2

| Parameter | N | Mean | Standard  Deviation | 25% | 50% | 75% |
| --- | --- | --- | --- | --- | --- | --- |
| Constant | 5000 | 0.005 | 0.020 | -0.008 | 0.005 | 0.019 |
| $r_{t-1}$ | 5000 | 0.322 | 0.050 | 0.288 | 0.322 | 0.356 |
| $i_{t-1}$ | 5000 | 0.515 | 0.047 | 0.484 | 0.516 | 0.546 |
| $r_{t-2}$ | 5000 | 0.047 | 0.053 | 0.011 | 0.046 | 0.082 |
| $i_{t-2}$ | 5000 | 0.285 | 0.046 | 0.254 | 0.284 | 0.315 |
| $\sigma^{2}$ | 5000 | 0.105 | 0.006 | 0.101 | 0.105 | 0.109 |

Cluster 3

| Parameter | N | Mean | Standard  Deviation | 25% | 50% | 75% |
| --- | --- | --- | --- | --- | --- | --- |
| Constant | 5000 | 0.005 | 0.020 | -0.008 | 0.005 | 0.019 |
| $r_{t-1}$ | 5000 | 0.106 | 0.054 | 0.071 | 0.106 | 0.141 |
| $i_{t-1}$ | 5000 | 0.068 | 0.058 | 0.028 | 0.067 | 0.107 |
| $r_{t-2}$ | 5000 | 0.094 | 0.052 | 0.059 | 0.095 | 0.129 |
| $i_{t-2}$ | 5000 | 0.112 | 0.054 | 0.076 | 0.113 | 0.149 |
| $\sigma^{2}$ | 5000 | 0.129 | 0.012 | 0.120 | 0.127 | 0.136 |

Cluster 4

| Parameter | N | Mean | Standard  Deviation | 25% | 50% | 75% |
| --- | --- | --- | --- | --- | --- | --- |
| Constant | 5000 | 0.567 | 0.066 | 0.522 | 0.567 | 0.610 |
| $r_{t-1}$ | 5000 | 0.001 | 0.004 | -0.002 | 0.001 | 0.004 |
| $i_{t-1}$ | 5000 | 0.432 | 0.066 | 0.388 | 0.433 | 0.476 |
| $r_{t-2}$ | 5000 | 0.001 | 0.005 | -0.002 | 0.001 | 0.004 |
| $i_{t-2}$ | 5000 | 0.000 | 0.007 | -0.005 | 0.000 | 0.004 |
| $\sigma^{2}$ | 5000 | 0.009 | 0.001 | 0.009 | 0.009 | 0.009 |
