# Supplementary material for: Biosensor Approach to Psychopathology Classification
Source: PLoS Comput Biol. 2010 Oct 21;6(10):e1000966. doi: 10.1371/journal.pcbi.1000966 (PMC2958801; doi:10.1371/journal.pcbi.1000966)
Supplement: Table S8 — Relation between clusters based on all dyads and clusters based on only healthy trustees. This table shows that for dyads with healthy trustees, there is a concordance between their cluster assignments across the two clusterings: when we cluster all dyads and when we only cluster dyads with healthy trustees. Of all the healthy dyads which were assigned to Cluster 1 in the clustering based on all the dyads, 100% got assigned to Cluster 1 in the healthy-trustees-only clustering. Of all the healthy dyads which were assigned to Cluster 2 in the clustering based on all the dyads, 91.9% got assigned to Cluster 2 in the healthy-trustees-only clustering. Of all the healthy dyads which were assigned to Cluster 3 in the clustering based on all the dyads, 95.0% got assigned to Cluster 3 in the healthy-trustees-only clustering. Of all the healthy dyads which were assigned to Cluster 4 in the clustering based on all the dyads, 89.5% got assigned to Cluster 4 in the healthy-trustees-only clustering. (0.02 MB DOC) [file pcbi.1000966.s014.doc]

Cluster number in the Percentage of dyads assigned to the same cluster classification based on in the classification based on only healthy trustees all dyads

1 100.0%

2 91.9%

3 95.0%

4 89.5%
