# Supplementary material for: Biosensor Approach to Psychopathology Classification
Source: PLoS Comput Biol. 2010 Oct 21;6(10):e1000966. doi: 10.1371/journal.pcbi.1000966 (PMC2958801; doi:10.1371/journal.pcbi.1000966)
Supplement: Table S9 — Clustering based on the investment ratios vs. clustering based on the return ratios. For clustering based on the investment ratios, different optimization techniques lead to the same optimal number of clusters K = 4. For clustering based on the investment ratios, different optimization techniques lead to different numbers of clusters: K = 2 and K = 5. For each of the corresponding three clusterings, for each of the four pathologies, we list of largest over-representation and, in parenthesis, the number of the cluster in which this over-representation occurs. A blank slot means that the corresponding pathology is not over-represented in any of the clusters. According to our computations (see Methods section), only over-estimations of 1.5 and larger are statistically significant. For clustering based on the investment ratios, all overrepresentations are statistically significant, and different pathology groups are overrepresented in different clusters - i.e., this clustering provides a statistically significant separation of different pathologies. For clusterings based on the return ratios, not all overrepresentations are statistically significant, and some groups are overrepresented in the same cluster. (0.02 MB DOC) [file pcbi.1000966.s015.doc]

Group Over-representation Over-representation Over-representation based on $i_{j}$ based on $r_{j}$ based on $r_{j}$ (case of 2 clusters) (case of 5 clusters)

ADHD 1.50 (1) -- 1.50 (5)

ASD 1.76 (2) 1.94 (1) --

BPD-M 2.85 (3) 1.47 (2) 2.31 (5)

BPD-N 1.69 (3) 1.19 (2) 1.23 (1)

MDD 2.55 (4) 1.58 (2) 1.55 (4)
